# Divergent action of fluoxetine in zebrafish according to responsivity to novelty

**DOI:** 10.1038/s41598-018-32263-y

**Published:** 2018-09-17

**Authors:** Débora Fior, Fernanda Dametto, Michele Fagundes, João Gabriel Santos da Rosa, Murilo Sander de Abreu, Gessi Koakoski, Renan Idalencio, Heloísa Helena de Alcântara Barcellos, Angelo Piato, Leonardo José Gil Barcellos

**Affiliations:** 1 Programa de Pós-Graduação em Bioexperimentação, Universidade de Passo Fundo (UPF), BR 285, São José, Passo Fundo, RS 99052-900 Brazil; 2 Universidade de Passo Fundo (UPF), BR 285, São José, Passo Fundo, RS 99052-900 Brazil; 3 Programa de Pós-Graduação em Farmacologia, Universidade Federal de Santa Maria (UFSM), Av. Roraima, 1000, Cidade Universitária, Camobi, Santa Maria, RS 97105-900 Brazil; 4 Laboratório de Psicofarmacologia e Comportamento (LAPCOM), Programa de Pós-Graduação em Neurociências, Instituto de Ciências Básicas da Saúde, Universidade Federal do Rio Grande do Sul (UFRGS), Av. Sarmento Leite 500/305, Porto Alegre, RS 90050-170 Brazil; 5 Programa de Pós-Graduação em Ciências Ambientais, Universidade de Passo Fundo, (UPF), BR 285, São José, Passo Fundo, RS 99052-900 Brazil

## Abstract

Here we show that the novel object recognition test can discriminate between high (HRN, neophobic) and low (LRN, neophilic) novelty responders in zebrafish populations. Especially when we observe the latency to the first entry in the novel object zone, zebrafish did not maintain these behavioral phenotypes in sequential tests and only the HRN group returned to their initial responsive behavior when exposed to fluoxetine. Our results have important implications for behavioral data analysis since such behavioral differences can potentially increase individual response variability and interfere with the outcomes obtained from various behavioral tasks. Our data reinforce the validity of personality determination in zebrafish since we show clear differences in behavior in response to fluoxetine.

## Introduction

Zebrafish (*Danio rerio*) are small and robust fish with a fast embryonic/larval development and are easily maintained in high quantities^1^. Due to these characteristics and their high genetic homology with humans^2^, this model organism is widely used in a variety of research fields, such as neuroscience, pharmacology, and genetics^3,4^. The evaluation of the zebrafish behavioral repertoire has become a strong research tool for biomedical research^5^. This species presents complex behavior and relative social interactions. In recent years, the behavior of this model organism has been elucidated and studied in many protocols. Zebrafish have proved to be an adequate model to study the effects of acute and chronic stress^6–10^ as well as anxiety and prey-predator relationships^11–13^. Similarly, the effects of various pharmaceutical agents and drugs on zebrafish behavior have been studied^14^.

Temperament can affect the ways in which animals interact with the environment, predators, food sources, and socially or sexually with members of the same species^15^. Temperament is frequently associated with responses to novel, risky or challenging situations^16^. Neophobia is a personality trait defined by the avoidance of novelty^14^. Understanding the differences between individualized behaviors facilitates the effective measurement of such responses^17^. In rodents, the behavioral phenotypes determined by individual temperament can impact behavioral testing outcomes^18^. Here we hypothesized that this also occurs in zebrafish. Previous studies have reported that shy zebrafish show low exploratory behavior^14^, while bold fish are highly explorative and increase their interactions with novel objects^15^.

Recently, our group demonstrated that fluoxetine (FLU), a serotonin modulator, affects endocrine^19,20^ and behavioral^21^ responses to stress. Serotonin is a neurotransmitter involved in behavioral pathways and is responsible for regulating individual responses to environmental demands^22^. Some studies have reported an association between zebrafish behavioral patterns and serotonin^23–25^. Thus, here we evaluate individual zebrafish according to their reactivity to novelty and investigate whether FLU modulates these personality traits.

## Results

**Figure 1:**
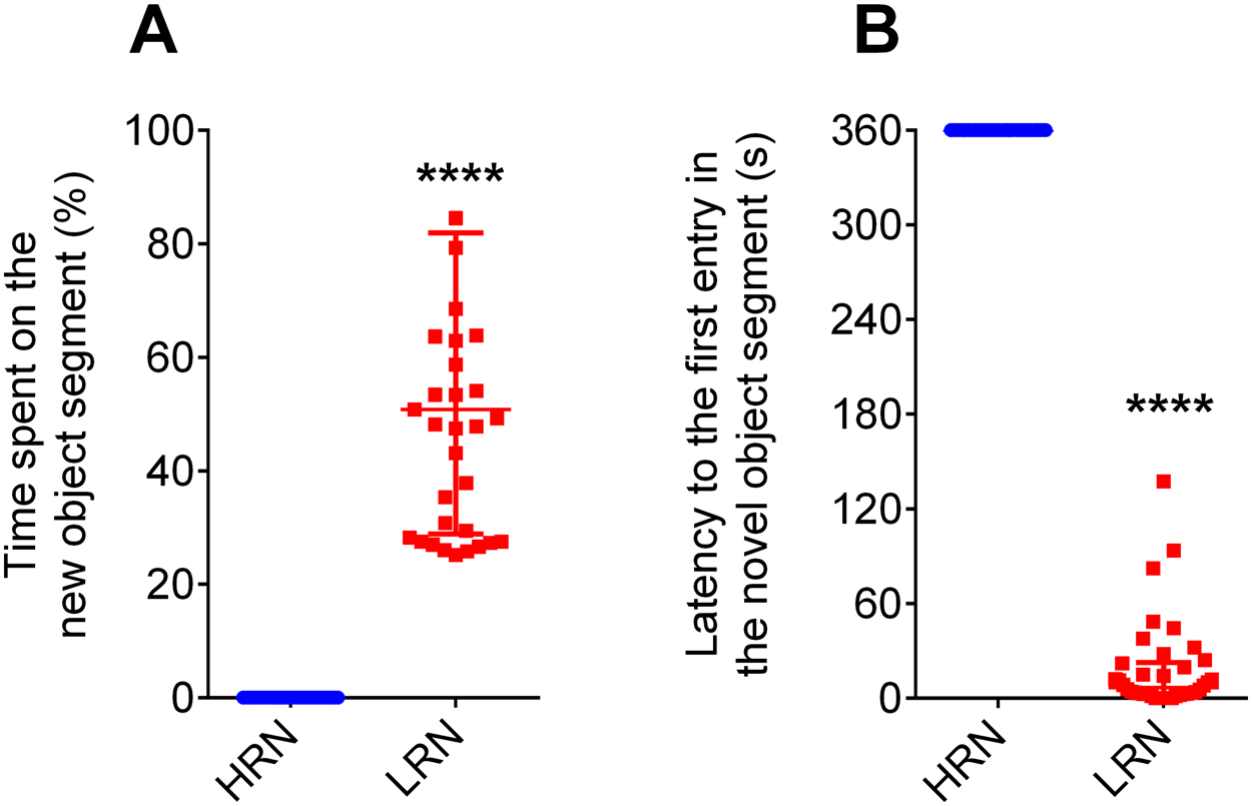
Time spent in (**A**) and latency to the first entry in the novel object zone (**B**) in the initial separation test. HRN = Fish high responders to novelty and LRN = Fish low responders to novelty. Data were presented as median ± interquartile range and analyzed by Kruskal-Wallis followed by Dunn test (****p < 0.0001).

In our initial novel objecting recognition (NOR) testing, 38 zebrafish that did not explore the novel object were classified as high responders to novelty (HRN, neophobic) and 38 zebrafish that spent more time around the novel object, as low responders to novelty (LRN, neophilic) (Fig. 1A).

**Figure 2:**
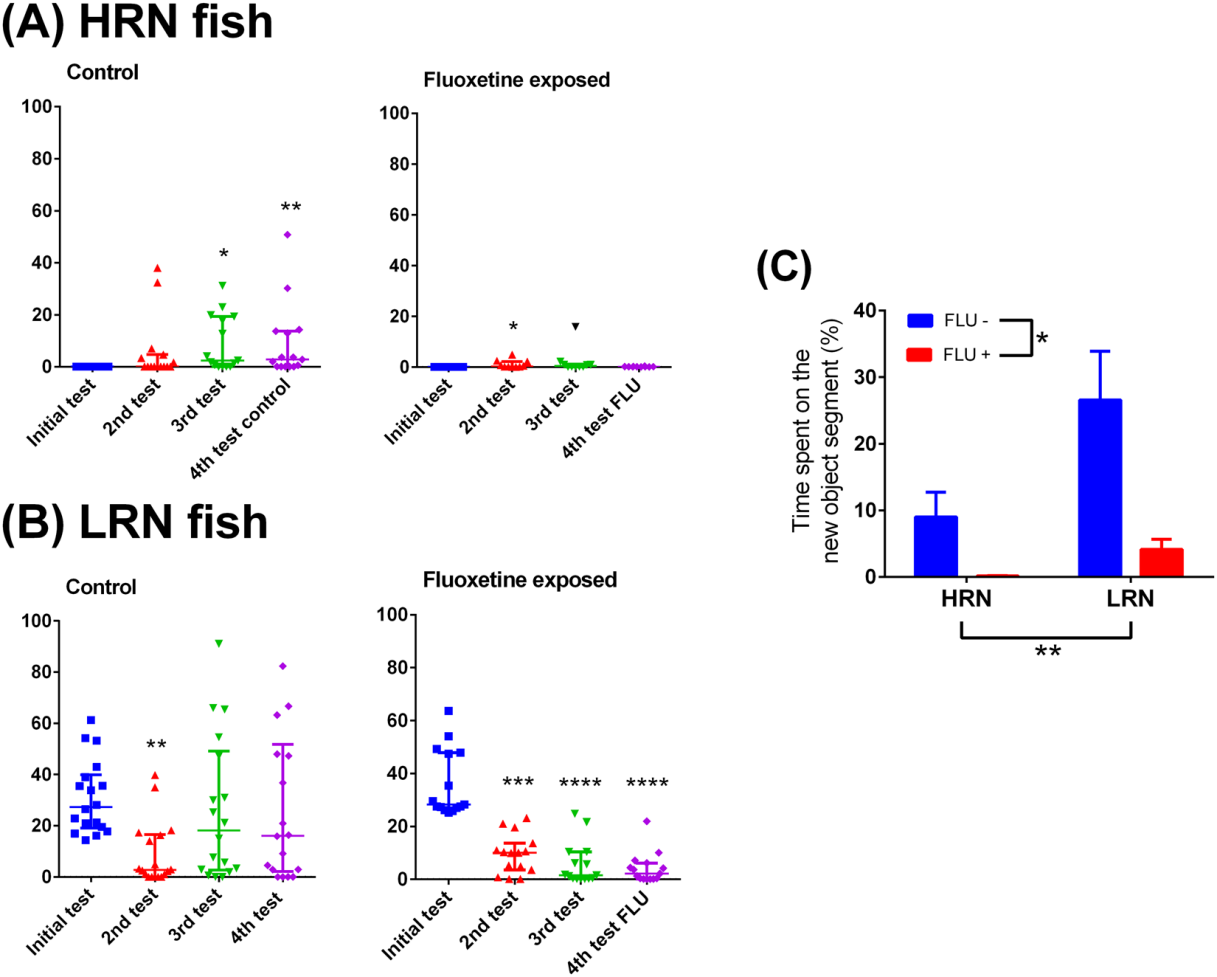
Relative time spent in the novel object zone. Fish high responders to novelty (HRN, **A**) and fish low responders to novelty (LRN, **B**). (**C**) Shows the two-way ANOVA of the 4^th^ test. Data in (**A**) and (**B**) are presented as median ± interquartile range and analyzed by Friedman’s test followed a post hoc analysis with Dunn’s test (*p < 0.05, **p < 0.01, ***p < 0.001 and ****p < 0.0001).

In the second test, the HRN fish lost their NOR responsiveness pattern and increase the time spent in the novel object segment. In fact, in the first test the fish did not explore the new object at all, but in the second trial, all animals visited the novel object at least once, although for a short period. HRN fish acutely exposed to FLU did not visit the novel object at any time whereas HRN unexposed to FLU showed an increase in time spent in the novel object zone. In contrast to HRN fish, LRN fish showed a reduction in time spent in the novel object zone in test 2 which returned to baseline in tests 3 and 4. Acute exposure to FLU had no effect on time spent in the novel object zone for LRN fish. Two-way ANOVA showed no interaction between factors (F_1,51_ = 2.133, *p* = 0.1503) but significant main effects of both personality (F_1,51_ = 11.33, *p* = 0.0015) and drug (F_1,51_ = 5.360, *p* = 0.0247) were observed (Fig. 2C).

**Figure 3:**
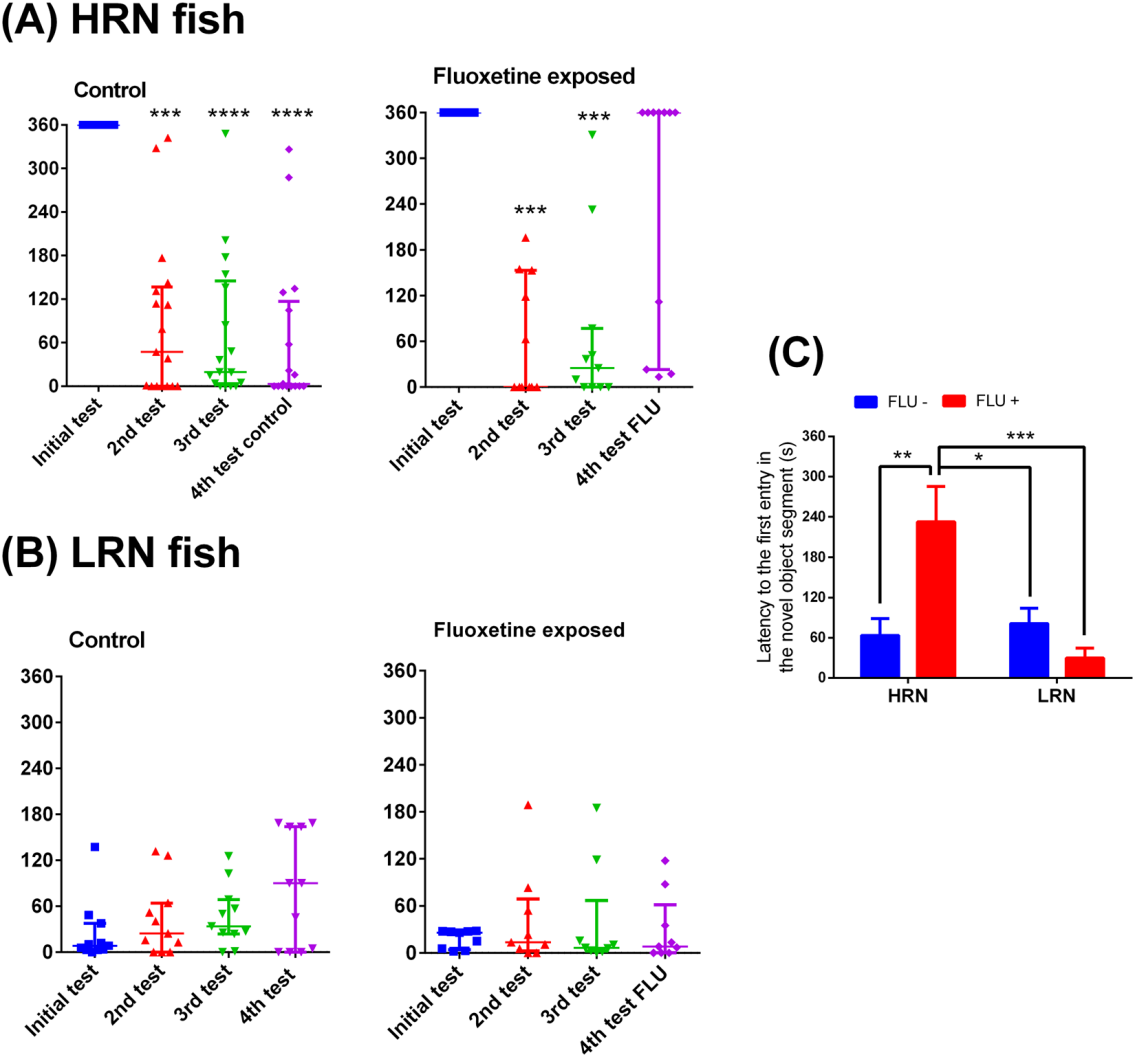
Latency to the first entry in the zone with the novel object. Fish high responders to novelty (HRN, **A**) and fish low responders to novelty (LRN, **B**). (**C**) Shows the two-way ANOVA of the 4^th^ test. Data in (**A**) and (**B**) are presented as median ± interquartile range and analyzed by Friedman’s test followed by Dunn’s test post hoc (*p < 0.05, **p < 0.01, ***p < 0.001 and ****p < 0.0001).

As expected, the 19 HRN fish presented higher latency to the first entry in the novel object segment than 19 LRN ones (Fig. 1B). In the subsequent tests, the HRN fish presented lower first-entry latencies (Fig. 3A). When acutely exposed to FLU, The HRN fish restore its initial behavior and the latency to the first entry in the novel segment object is statistically indistinguishable from the initial test level (Fig. 3A). No changes were observed in LRN fish in the sequential tests (Fig. 3B). Two-way ANOVA showed interaction between factors (F_1,43_ = 11.78, *p* = 0.0013) (Fig. 3C). The HRN fish exposed to FLU presented higher latency than other groups.

**Figure 4:**
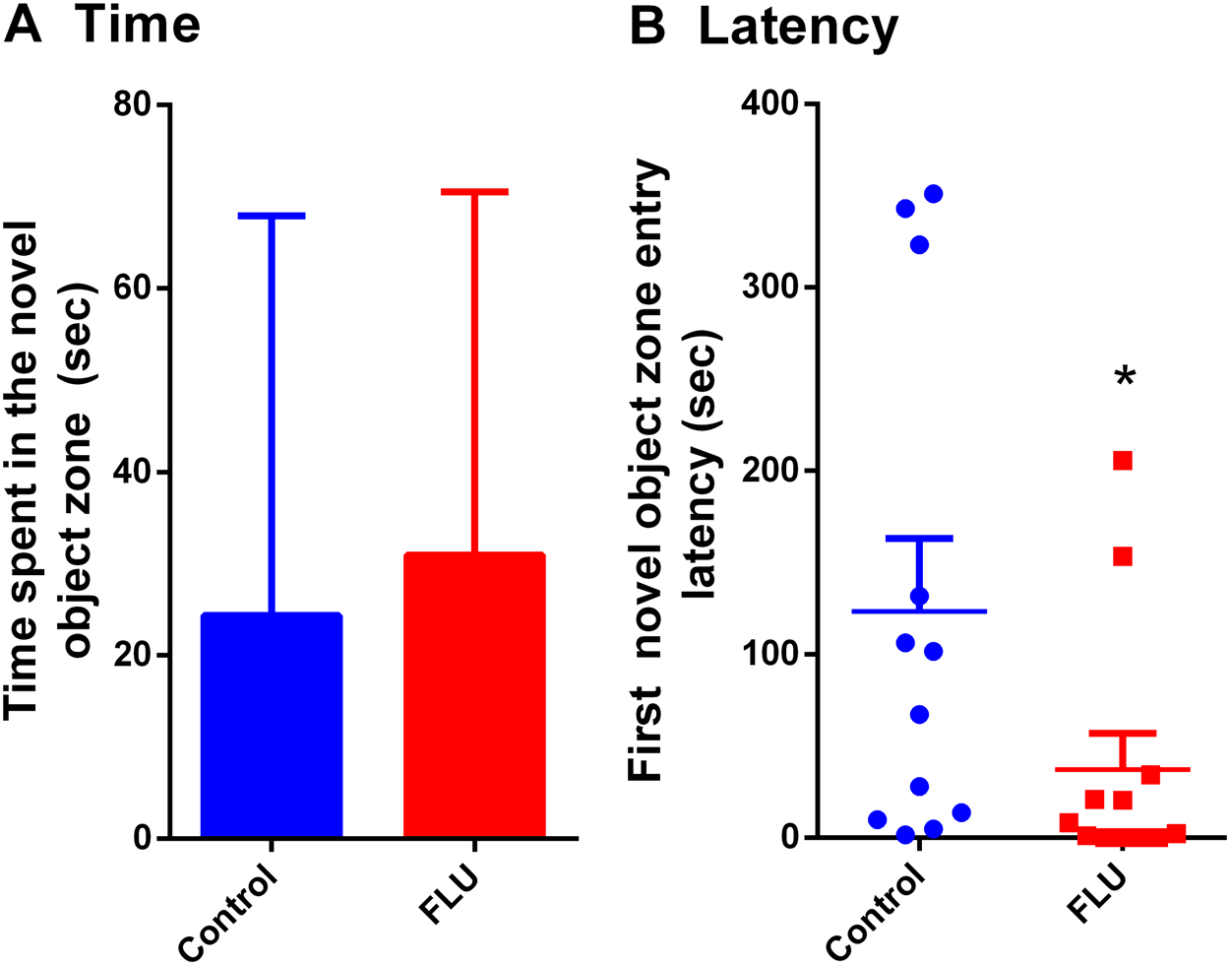
Exploration time and visit latency in the novel object zone. Time spent in the novel object zone (**A**) and latency to the first entry in the novel object zone (**B**) in the unified zebrafish population. In panel A, data are presented as mean + SD and analyzed by Student’s t-test, while in panel B data are represented as median ± interquartile range and analyzed by Mann Whitney test post hoc (**p < 0.01).

In the unified zebrafish population, FLU exposure did not affect the time spent in the novel object zone (Fig. 4A) but significantly decreased the first novel object zone entry latency (Fig. 4B).

**Figure 5:**
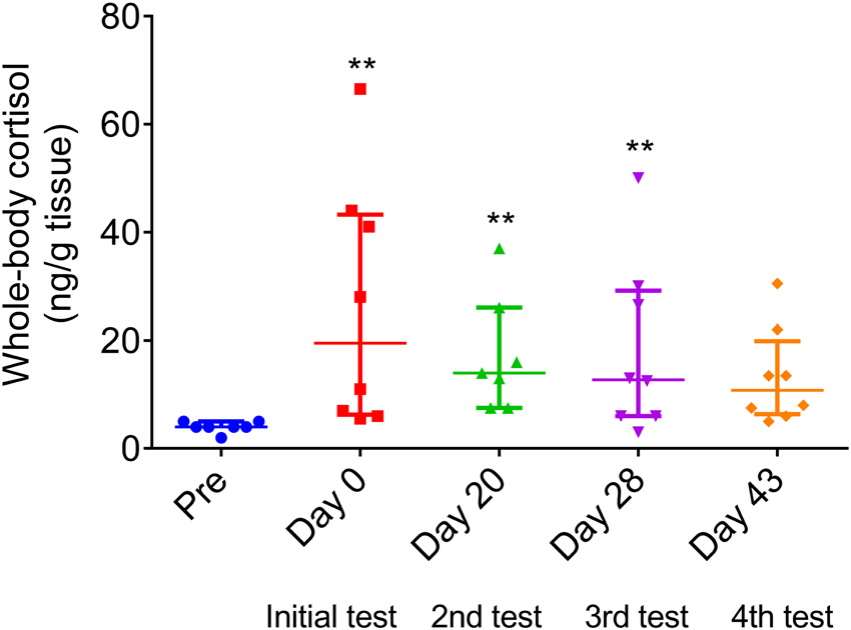
Whole-body cortisol levels during the 43-day period of the individualized housing. Data are presented as median ± interquartile range and analyzed by Kruskal-Wallis followed by Dunn’s test post hoc (**p < 0.01).

Regarding cortisol levels, the average concentration in the unified zebrafish population prior to experimental procedures was 4 ± 0.38 ng/g of wet tissue. The netting from the aquarium and their transportation to individualized housing significantly increased cortisol levels. After 20 and 28 days (2^nd^ and 3^rd^ test), whole-body cortisol levels were still higher than the concentrations detected in pre-experimental zebrafish. Finally, after 43 days of individualized housing (4^th^ test), cortisol decreased to basal pre-experimental levels (Fig. 5).

**Figure 6:**
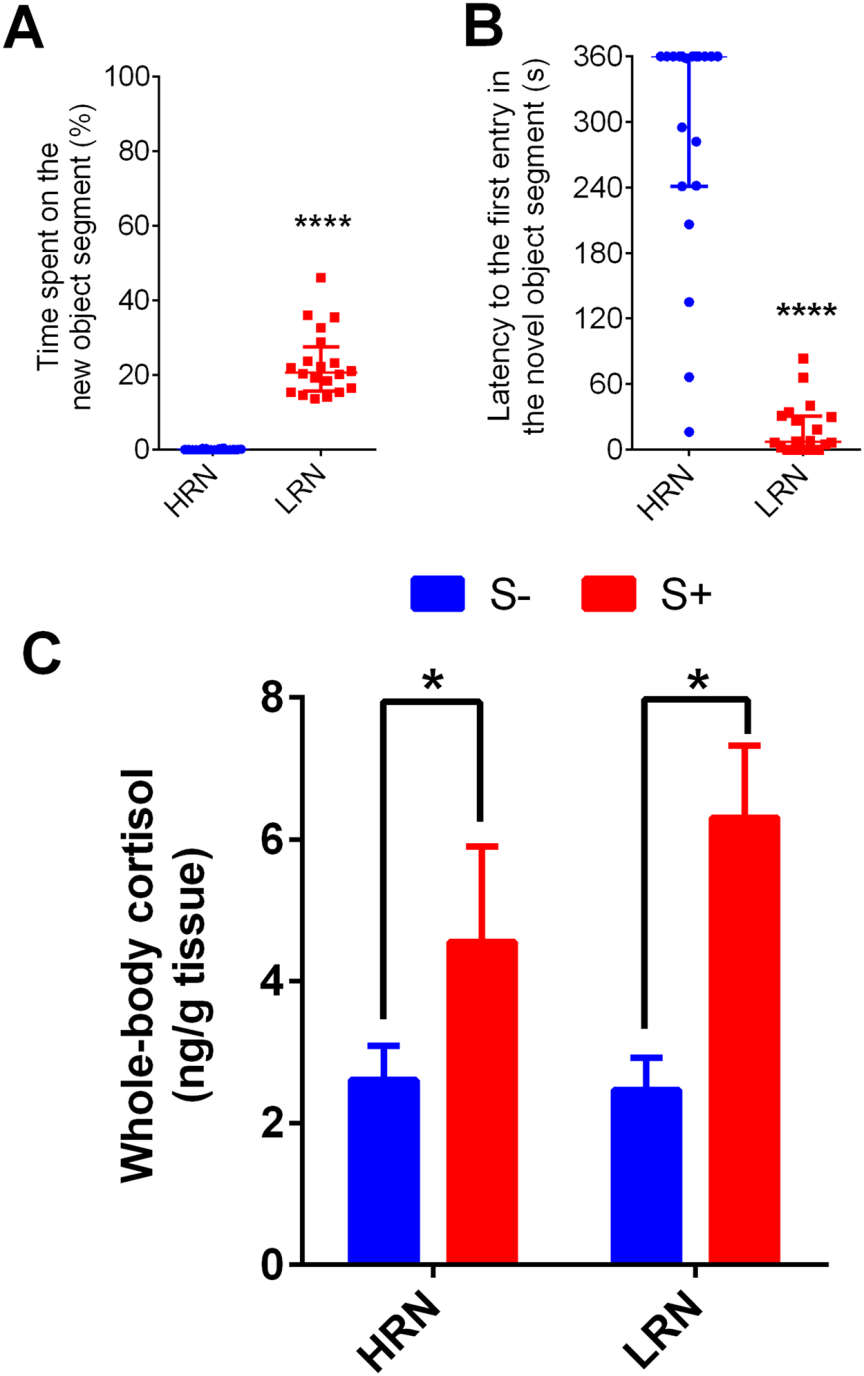
Time spent in the novel object segment (**A**) and latency to the first entry in the novel object segment (**B**) in fish high and low responders to novelty (HRN and LRN, respectively); and (**C**) two-way ANOVA of stress responsiveness test. Data in **A** and **B** are presented as median ± interquartile range and analyzed by Mann Whitney test. Data in **C** are presented as mean ± S.E.M. and analyzed by two-way ANOVA followed by Tukey’s multiple range test. (*p < 0.05 and ****p < 0.0001).

In the stress responsiveness experiment, both HRN and LRN were separated according to the time spent near to the novel object and the latency to the first entry in the new object segment (Fig. 6A and B, respectively). We separated 20 HRN fish, 10 exposed to blue and 10 to green new objects. Similarly, LRN group of 20 fish resulted in exposure to blue (11 fish) and green (9 fish) new objects. No differences were found in the time spent near to the novel object between both colors (please see Table 8 of Supplementary material). Regarding whole-body cortisol levels, no interaction was found between fish responsiveness to novelty and acute stress (F_1,36_ = 1.083, *p* < 0.3050). There was significant effect of stress (F_1,36_ = 10.16, *p* < 0.0030) (Fig. 6C), and no effect of fish personality (F_1,36_ = 0.7859, *p* < 0.3812).

## Discussion

Here we show that the NOR protocol was able to discriminate between zebrafish with high (HRN, neophobic) and low (LRN, neophilic) responses to novelty. Observing the latencies to the first novel object inspection, we also demonstrate that zebrafish did not maintain these behavioral phenotypes in sequential testing (Fig. 3). In addition, we show that only HRN fish returned to their initial responsiveness behavior after exposure to fluoxetine.

The NOR protocol used in the present study has been previously used to distinguish HRN from LRN fish^26^. In zebrafish, the NOR test has also been used to evaluate learning and memory^27–29^. This test has been extensively used to evaluate explorative and attentive behaviors induced by the presence of a novel stimulus^30^, in humans^31^, primates^32^ and rodents^33^.

Our data demonstrate diverse zebrafish responsiveness behaviors in new situations (i.e., novel object), with shy and bold behavioral phenotypes, as previously reported^15,34^. These behavioral patterns are important, since such characteristics may directly interfere with experimental outcomes in various behavioral tasks.

The most studied *continuum* in zebrafish is the “shy-bold” behavioral variation. In this *continuum*, bold fish generally present high explorative activity towards novel objects, reduced anxiety-like behaviors and changes in body color^15,34^. In contrast, shy fish demonstrate low exploratory activity and increased reactivity to novelty and risk^14^. Other studies have investigated the genetic base of such “shy-bold” behavioral patterns and have stipulated that the bold zebrafish phenotype may present with a genetic evolutionary advantage^35^. In fact, bold and aggressive personality traits have been shown to be heritable with a significant maternal inheritance proportion^36^.

Observing the latency to the 1^st^ inspection of the new object, we show that the initial HRN and LRN behavior were not maintained in the second NOR test (Fig. 3). Despite the color of the novel object was randomly chosen, all HRN fish were presented with a green novel object. Interestingly, when these fish were tested again, we used blue novel objects and the HRN behavioral characteristic was lost (Fig. 3). However, in the second trial (HPI responsiveness experiment), color did not affect the responsivity to novelty. Some studies have shown that larval and adult zebrafish have a preference for blue color^37–39^; instead of green^38,40^. Thus, we cannot exclude the possibility that the color of the novel object may have influenced the neophobic responses.

However, this hypothesis loses power when we analyze data from the HPI responsiveness experiments where half the fish were presented with blue, and half with green, new objects and react in exactly the same manner.

Another hypothesis that might explain the loss of the initial responsiveness in HRN and LRN zebrafish, is the effect of stress caused by fish isolation^41^, a strategy used in our study to verify the identity of each fish. Reinforcing this hypothesis, the isolation apparatus seemed to induce stress in zebrafish (Fig. 5). Over time, zebrafish adapted to this apparatus and whole-body cortisol levels returned to pre-experimental levels (Fig. 5). Such attenuation of the neuroendocrine stress axis has been previously described in several fish species like the Nile tilapia^42^, the Siluridae, *Rhamdia quelen*^43^, as well as in zebrafish^6^. In addition, the reversal of cortisol to basal levels could be contributed to the return of the initial responsiveness (1^st^ test, based on the latency to explore novel object) in HRN fish (Fig. 3). Reinforcing this hypothesis, both HRN and LRN activates their HPI axis in the same manner when challenged by an acute stressor (Fig. 6C). Thus, we demonstrated the association of stress (increase in cortisol levels) with behavioral responsiveness, since stress (e.g., chasing) is known to cause behavioral changes, such as increased anxiety in zebrafish^21^.

Interestingly, despite the loss of the initial behavioral pattern (Fig. 3), HRN fish returned to their initial responsiveness phenotype when exposed to FLU, prior to the test. In contrast, HRN fish that were not exposed to FLU, followed the same pattern observed in the 2^nd^ and 3^rd^ test (Fig. 3). This link between novelty responsiveness and FLU exposure was reinforced by the data from two-way ANOVA showing an interaction between personality and FLU in relation to the latency to the first entry in the novel object segment and main effects of personality and FLU exposure, in relation to the time spent near to the novel object. Our hypothesis is that the isolation period (43 days) might have induced a hippocampal dysfunction that consequently led to a reduction in serotoninergic activity, as previously detected in rodents^44^ and in zebrafish^45^.

Several fish behaviors have been correlated with serotonin levels and, consequently, drugs that act via serotoninergic pathways, such as FLU, may modulate behavioral phenotypes^23^. Serotonin is known to modulate locomotion and motor functions in zebrafish^46^. When used in stressful conditions, FLU demonstrates a clear anxiolytic effect, with a reduction in cortisol concentrations^19^ and subsequent modulation of anxiety-like social behaviors^21^. However, acute FLU administration in conditioned fear settings is anxiogenic^47^ in zebrafish and such a paradoxical effect has also been observed after acute FLU administration in humans^48^. The possible anxiogenic effect of acute FLU administration after the 43-day isolation may explain the restoration of responsiveness in HRN fish, especially related to the latency to the first novel object inspection. In addition, serotonin seems to have a dual or bi-directional effect on zebrafish behavior causing divergent responses to serotoninergic drugs in the novel tank and light-dark tests^25^. Variations in serotoninergic activities observed in fish might be induced by individual changes in anxiety levels^24^. The modulation of bold behavior by FLU has been observed in both male and female *Betta splenden*^49,50^.

No differences in locomotor parameters between personality patterns were found, reinforcing the direct effect of FLU on the reactivity to novelty and not as a consequence of changes in fish locomotion (see Supplementary material).

Interestingly, exposure to FLU in the unified zebrafish population (not classified as HRN or LRN) did not affect the time spent in the novel object zone (Fig. 3A). However, FLU exposure decreased the first novel object zone entry latency (Fig. 3B). The very high result variability (high SDs) observed in this study, reinforces the idea that different behavioral phenotypes can influence testing outcomes.

A possible limitation of this study is our inability to investigate the precise mechanisms by which FLU modulates responsiveness. However, this does not lessen the importance of this initial evaluation, since behavioral data regarding the effects of FLU in different zebrafish personalities are scarce.

Finally, the possible implications of behavior and personality discrimination patterns should be considered. These behavioral patterns can increase the variability of individual zebrafish responses and interfere with the outcomes obtained from various behavioral tasks used in scientific research. In fact, personality traits can affect the interaction patterns between individuals^15^. In zebrafish^51^, and in guppies, *Poecilia reticulata*^52^ several individual differences have already been described. Thus, our data verify the existence of personality determination and discrimination in zebrafish, since we show clear behavioral differences following FLU administration.

## Material and Methods

### Ethical note

The study was approved by the Ethics Commission for Animal Use at Universidade de Passo Fundo, Brazil (Protocol 31/2016-CEUA) and fully complied with the guidelines of Conselho Nacional de Controle de Experimentação Animal, Brazil (CONCEA).

### Animals and housing conditions

A population of 450 mixed sex (50:50), adult, wild-type, zebrafish of short-fin (SF) phenotype, weighing 0.3–0.5 g from a single breeding, were held in a tank equipped with biological filters, with a natural photoperiod (14 h light:10 h dark cycles) and under constant ventilation. Fish were fed twice a day, with commercial flaked food provided *ad libidum*. Water temperature was maintained at 28 ± 2 °C, dissolved oxygen concentrations at 6.1 ± 0.2 mg/L, pH 7.0 ± 0.2, and the total ammonia concentration was less than 0.5 mg/L. Fish used in this study were in perfect health and were not subjected to any other procedure or drug exposure prior to the experiments.

### Study strategy

**Figure 7:**
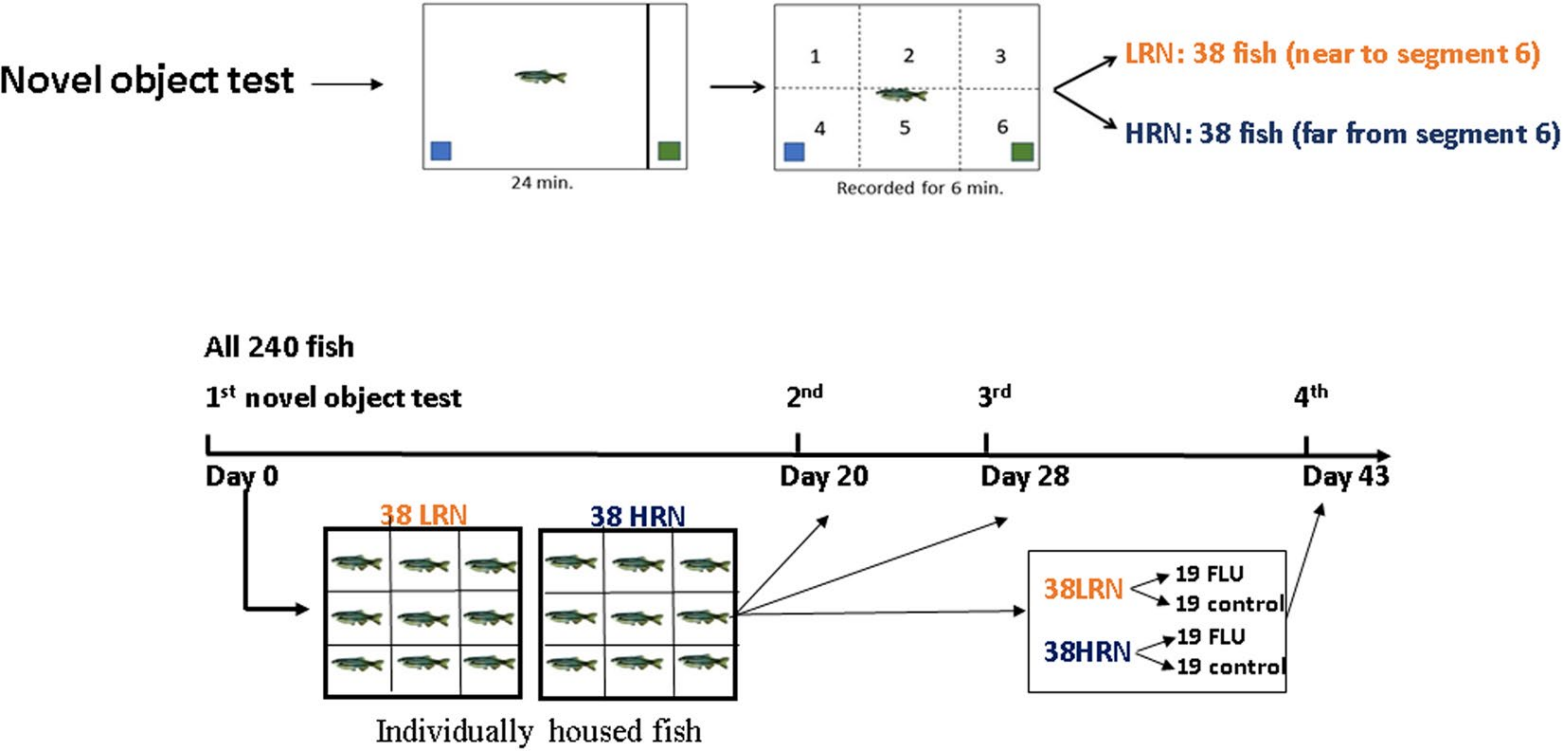
Study design. Schematic representation of the study plan. LRN (low responders to novelty); HRN (high responders to novelty); FLU (fluoxetine). The fish were drawn by LJGB.

Our study strategy was to evaluate the responsiveness of zebrafish when submitted to a novel object. Fish were separated into two groups, the high (HRN, neophobic) and the low novelty responders (LRN, neophilic). In our first test, we considered as HRN the fish that did not explore the novel object at any time during the entire test and LRN fish, the ones that spent more time in the novel object zone, as described by Kirsten *et al*.^26^. After our initial screening, we housed fish individually, after which all HRN and LRN fish were submitted to three more NOR tests. In the fourth NOR test, half of the zebrafish from the HRN and LRN groups were exposed to FLU prior to testing, while the rest of the HRN and LRN groups were evaluated without FLU exposure (see schematic representation in Fig. 7).

In order to verify the effect of FLU in the general population (without separating HRN and LRN), and the HPI axis responsiveness in HRN and LRN separated populations, we performed two additional studies. In the first, we tested 12 control and 12 FLU-exposed fish in the NOR test, while in the second we separated a population of 90 adult zebrafish into two groups, the high (HRN, neophobic) and the low novelty responders (LRN, neophilic). As made in the main experiment, we considered as HRN the fish that did not explore the novel object at any time during the entire test and LRN fish, the ones that spent more time in the novel object zone, as described by Kirsten *et al*.^26^. After this initial separation test, we performed an acute stress test (as described Giacomini *et al*.^21^) in a half of fish of each personality pattern and measured the whole-body cortisol as the indicator of HPI responsiveness.

## Procedures

### NOR test, individual housing apparatus, and sequential testing

The protocol used was adapted from Braida *et al*.^30^ and May *et al*.^28^. Briefly, in a glass aquarium (24 × 8 × 20 cm) we inserted an object in each aquarium side. These objects were plastic cubes (with 4 cm lateral size and blue or green color). We separated the novel object zone (about one-third of the aquarium) from the rest of the aquarium with an opaque partition, aiming at restricting the zebrafish from seeing the novel object, as previously described by Kirsten *et al*.^26^ Zebrafish were individually introduced in the open area (about two-thirds of the aquarium) for 24 minutes to habituate to the presence of the object^30^ (based on the time for memory acquisition^27^).

After this period, the opaque partition was removed, and the fish were able to see another object (“novel object”). The reaction of the fish to the presence of this novel object was filmed for 6 minutes, using a Logitech HD Webcam C525 camera located in the front of the tank. The videos were then analyzed offline using the ANY-maze^®^ software. The test tank was divided into six virtual zones, and the following behavioral parameters were scored: (a) the time spent in the novel object zone and (b) the latency for the first entry in this zone.

In the first testing round, 240 fish were evaluated and, then each individual fish was kept isolated in one of the 24 compartments of a 100 L plastic tank. In this individualized apparatus, fish maintain chemical and visual contact but did not form a shoal. For this reason, we also evaluated the stress-inducing potential of this apparatus.

Twenty days after the 1^st^ test, 38 HRN and 38 LRN fish were tested again to verify if the high and low responses to novelty were maintained. Eight days after the 2^nd^ test, 76 fish were retested inverting the color of the novel object, aiming to evaluate if color preference can interfere with behavioral responses.

Fifteen days later, the 4^th^ test aimed to evaluate if FLU exposure (50 μg/L, Darin® EMS São Paulo, Brazil) for 15 minutes prior to NOR testing, modulated the HRN and LRN behavioral responses to novelty. Each fish was captured and exposed to FLU individually, in a beaker, prior to their introduction to the novel object. The control group underwent the same procedure without FLU in the water and was performed separately.

Each group of fish tested in the 4^th^ test is formed by the same fish from the 1^st^ test to data pairing to data analysis that accommodates the lack on independence between groups formed by the same individuals (repeated measures ANOVA or Friedmann’s test depends on data normality).

For the FLU test in the unified zebrafish population, we used 24 fish distributed in two groups, one group was exposed to FLU (the same protocol as described above) and the second was the control group.

### Whole-body cortisol determination additional experiments

For whole-body cortisol determination, we performed an additional experiment using 40 fish from the stock population. From these 40 fish, 8 were killed to determine the basal pre-experimental cortisol concentration. The remaining 32 fish were then distributed into four groups to simulate the exact periods of the NOR tests (0, 20, 28 and 43 days, corresponding to initial, 2^nd^, 3^rd^ and 4^th^ tests). In the HPI responsiveness experiment, we evaluated the cortisol response to an acute stressor in fish of both personality patterns. In both experiments, we assessed whole-body cortisol concentration in zebrafish, extracted and determined using the method described previously^11^.

### Statistics

Since fish were individualized and the same in each sequential testing and data did not meet the premises for parametric tests, we compare time and latency data using the Friedman’s test, which is analogous to the repeated measures ANOVA, followed by a Dunn’s test post-hoc. Additionally, restricted to the fourth test, we compare control and flu-exposed fish of both HRN and LRN groups by a two-way ANOVA with flu exposure and fish personality as independent factors. To compare cortisol data, we used one-way ANOVA followed by a Dunn’s post-hoc test since data did not present normal distribution (assessed by the Kolmogorov-Smirnov test). The unpaired *t*-test or Mann-Whitney U test were used to analyze the initial testing and the non-classified fish NOR test results. In the HPI responsiveness experiment, we evaluated the cortisol response to an acute stressor in a stressed and control fish of both personality patterns by two-way ANOVA with personality and stress as independent factors. Significance was set at *P* < 0.05 in all analyses.

## Electronic supplementary material

Dataset 1
