# Supplementary material for: Divergent action of fluoxetine in zebrafish according to responsivity to novelty
Source: Sci Rep. 2018 Sep 17;8:13908. doi: 10.1038/s41598-018-32263-y (PMC6141609; doi:10.1038/s41598-018-32263-y)
Supplement: Supplementary file 1 — Dataset 1 [file 41598_2018_32263_MOESM1_ESM.doc]

Supplementary information – data set

**Divergent action of fluoxetine in zebrafish according to responsivity to novelty**

Débora Fior, Fernanda Dametto, Michele Fagundes, João Rosa, Murilo Abreu, Gessi Koakoski, Renan Idalencio, Heloísa Barcellos, Angelo Piato, Leonardo José Gil Barcellos

**Table 1. Data from Figure 1A Time spent in the novel object zone, Initial separation test**

| HRN | LRN |
| --- | --- |
| 0 | 26.99 |
| 0 | 27.34 |
| 0 | 27.54 |
| 0 | 25.85 |
| 0 | 25.20 |
| 0 | 47.85 |
| 0 | 27.49 |
| 0 | 29.49 |
| 0 | 35.34 |
| 0 |  |
| 0 | 28.25 |
| 0 | 26.06 |
| 0 | 37.83 |
| 0 | 54.07 |
| 0 | 26.62 |
| 0 | 49.27 |
| 0 | 47.46 |
| 0 | 63.67 |
| 0 | 53.38 |
| 0 | 53.40 |
| 0 | 116.80 |
| 0 | 43.10 |
| 0 | 79.30 |
| 0 | 101.40 |
| 0 | 106.80 |
| 0 | 128.80 |
| 0 | 84.50 |
| 0 | 68.50 |
| 0 | 48.20 |
| 0 | 162.80 |
| 0 | 58.70 |
| 0 | 106.40 |
| 0 | 62.90 |
| 0 | 50.80 |
| 0 | 159.60 |
| 0 | 183.90 |
| 0 | 63.80 |
| 0 | 30.80 |

| Unpaired t test |  |
| --- | --- |
| P value | < 0,0001 |
| P value summary | **** |
| Significantly different? (P < 0.05) | Yes |
| One- or two-tailed P value? | Two-tailed |
| t, df | t=9,339 df=73 |

**Table 2. Data from Figure 1B Latency to the first entry in the zone with the novel object, initial separation test**

| HRN | LRN |
| --- | --- |
| 360.0 | 5.50 |
| 360.0 | 2.60 |
| 360.0 | 4.30 |
| 360.0 | 2.10 |
| 360.0 | 28.00 |
| 360.0 | 15.00 |
| 360.0 | 1.50 |
| 360.0 | 0.00 |
| 360.0 | 0.00 |
| 360.0 | 0.00 |
| 360.0 | 4.10 |
| 360.0 | 22.10 |
| 360.0 | 44.40 |
| 360.0 | 12.00 |
| 360.0 | 0.00 |
| 360.0 | 3.10 |
| 360.0 | 0.00 |
| 360.0 | 2.80 |
| 360.0 | 3.70 |
| 360.0 | 137.40 |
| 360.0 | 5.70 |
| 360.0 | 37.70 |
| 360.0 | 8.40 |
| 360.0 | 11.90 |
| 360.0 | 10.10 |
| 360.0 | 48.40 |
| 360.0 | 8.00 |
| 360.0 | 24.30 |
| 360.0 | 1.60 |
| 360.0 | 3.00 |
| 360.0 | 2.70 |
| 360.0 | 14.30 |
| 360.0 | 32.30 |
| 360.0 | 93.50 |
| 360.0 | 19.80 |
| 360.0 | 3.60 |
| 360.0 | 11.40 |
| 360.0 | 82.30 |

| Unpaired t test |  |
| --- | --- |
| P value | < 0,0001 |
| P value summary | **** |
| Significantly different? (P < 0.05) | Yes |
| One- or two-tailed P value? | Two-tailed |
| t, df | t=72,11 df=74 |

**Tables 3. Data from Figure 2 Time spent in the novel object zone**

1. **HRN**

Control

| Initial test | 2nd test | 3rd test | 4th test control |
| --- | --- | --- | --- |
| 0.1 | 4,700000 | 1.80 | 3.700 |
| 0.1 | 0,100000 | 0.70 | 12.900 |
| 0.1 | 1,700000 | 19.40 | 0.600 |
| 0.1 | 0,100000 | 0.10 | 30.200 |
| 0.1 | 3,300000 | 22.90 | 13.700 |
| 0.1 | 0,100000 | 1.40 | 0.100 |
| 0.1 | 0,100000 | 4.10 | 0.100 |
| 0.1 | 38,000000 | 12.70 | 0.100 |
| 0.1 | 0,100000 | 17.70 | 50.800 |
| 0.1 | 0,100000 | 0.10 | 14.300 |
| 0.1 | 7,000000 | 19.90 | 3.700 |
| 0.1 | 32,400000 | 2.40 | 2.800 |
| 0.1 | 0,100000 | 0.10 | 0.100 |
| 0.1 | 0,100000 | 31.20 | 2.000 |
| 0.1 | 0,100000 | 0.10 | 0.100 |

| Table Analyzed | HRN CTRL |
| --- | --- |
|  |  |
| Friedman test |  |
| P value | 0,0016 |
| Exact or approximate P value? | Approximate |
| P value summary | ** |
| Are means signif. different? (P < 0.05) | Yes |
| Number of groups | 4 |
| Friedman statistic | 15,25 |
|  |  |
| Data summary |  |
| Number of treatments (columns) | 4 |
| Number of subjects (rows) | 15 |

| Number of families | 1 |  |  |  |  |
| --- | --- | --- | --- | --- | --- |
| Number of comparisons per family | 3 |  |  |  |  |
| Alpha | 0,05 |  |  |  |  |
|  |  |  |  |  |  |
| Dunn's multiple comparisons test | Rank sum diff, | Significant? | Summary | Adjusted P Value |  |
|  |  |  |  |  |  |
| Initial test vs. 2nd test | -13,50 | No | ns | 0,1689 |  |
| Initial test vs. 3rd test | -22,00 | Yes | ** | 0,0056 |  |
| Initial test vs. 4th test control | -18,50 | Yes | * | 0,0267 |  |
|  |  |  |  |  |  |
|  |  |  |  |  |  |
|  |  |  |  |  |  |

Fluoxetine exposed

| Initial test | 2nd test | 3rd test | 4th test FLU |
| --- | --- | --- | --- |
| 0.1 | 2,140816 | 0.87 | 0.190 |
| 0.1 | 4,879233 | 0.10 | 0.100 |
| 0.1 | 0,152826 | 0.10 | 0.174 |
| 0.1 | 0,100000 | 0.88 | 0.100 |
| 0.1 | 0,125100 | 0.10 | 0.100 |
| 0.1 | 0,147962 | 15.86 | 0.390 |
| 0.1 | 0,100000 | 0.91 | 0.100 |
| 0.1 | 2,442787 | 0.10 | 0.100 |
| 0.1 | 0,435973 | 0.10 | 0.100 |
| 0.1 | 0,736780 | 2.16 | 0.168 |

| Table Analyzed | HRN FLU |
| --- | --- |
|  |  |
| Friedman test |  |
| P value | 0,0117 |
| Exact or approximate P value? | Approximate |
| P value summary | * |
| Are means signif. different? (P < 0.05) | Yes |
| Number of groups | 4 |
| Friedman statistic | 11,00 |
|  |  |
| Data summary |  |
| Number of treatments (columns) | 4 |
| Number of subjects (rows) | 10 |

| Number of families | 1 |  |  |
| --- | --- | --- | --- |
| Number of comparisons per family | 3 |  |  |
| Alpha | 0,05 |  |  |
|  |  |  |  |
| Dunn's multiple comparisons test | Rank sum diff, | Significant? | Summary |
|  |  |  |  |
| Initial test vs. 2nd test | -15,50 | Yes | * |
| Initial test vs. 3rd test | -12,00 | No | ns |
| Initial test vs. 4th test FLU | -6,500 | No | ns |

1. **LRN**

Control

| Initial test | 2nd test | 3rd test | 4th test FLU |
| --- | --- | --- | --- |
|  |  |  |  |
| 17.80000 | 3.20 | 47.30 | 108.900 |
| 38.93333 | 4.90 | 2.90 | 2.800 |
| 14.36667 | 17.30 | 54.60 | 20.900 |
| 26.43333 | 16.30 | 25.30 | 0.000 |
| 33.80000 | 0.00 | 0.60 | 2.900 |
| 35.60000 | 2.90 | 3.30 | 0.000 |
| 42.93333 | 0.00 | 0.00 | 9.100 |
| 28.16667 | 35.00 | 5.70 | 47.300 |
| 22.83333 | 18.20 | 30.00 | 66.700 |
| 16.06667 | 39.80 | 31.00 | 63.200 |
| 54.26667 | 14.00 | 1.90 | 48.000 |
| 19.56667 | 2.20 | 91.10 | 36.800 |
| 35.46667 | 2.50 | 7.60 | 82.400 |
| 20.96667 | 0.00 | 15.00 | 0.000 |
| 16.93333 | 1.00 | 21.20 | 0.000 |
| 53.20000 | 0.00 | 66.00 | 4.500 |
| 61.30000 | 0.00 | 65.40 | 16.300 |
| 21.26667 | 1.20 | 0.00 | 15.900 |

| Table Analyzed | LRN CTRL |
| --- | --- |
|  |  |
| Friedman test |  |
| P value | 0,0207 |
| Exact or approximate P value? | Approximate |
| P value summary | * |
| Are means signif. different? (P < 0.05) | Yes |
| Number of groups | 4 |
| Friedman statistic | 9,758 |
|  |  |
| Data summary |  |
| Number of treatments (columns) | 4 |
| Number of subjects (rows) | 18 |

| Number of families | 1 |  |  |
| --- | --- | --- | --- |
| Number of comparisons per family | 3 |  |  |
| Alpha | 0,05 |  |  |
|  |  |  |  |
| Dunn's multiple comparisons test | Rank sum diff, | Significant? | Summary |
|  |  |  |  |
| Initial test vs. 2nd test | 23,00 | Yes | ** |
| Initial test vs. 3rd test | 6,500 | No | ns |
| Initial test vs. 4th test | 6,500 | No | ns |

Fluoxetine exposed

| Initial test | 2nd test | 3rd test | 4th test FLU |
| --- | --- | --- | --- |
| 26.99000 | 10.96 | 1.72 | 0.100 |
| 27.34000 | 13.63 | 5.61 | 0.100 |
| 27.54000 | 23.16 | 10.38 | 7.150 |
| 25.85000 | 5.18 | 0.38 | 0.100 |
| 25.20000 | 0.10 | 0.22 | 3.630 |
| 47.85000 | 4.90 | 6.06 | 4.190 |
| 27.49000 | 10.63 | 0.17 | 0.314 |
| 29.49000 | 19.64 | 24.78 | 21.900 |
| 35.34000 | 10.30 | 21.59 | 6.140 |
| 28.25000 | 20.97 | 0.10 | 10.030 |
| 26.06000 | 0.10 | 0.10 | 4.400 |
| 54.07000 | 10.04 | 0.36 | 2.120 |
| 49.27000 | 0.74 | 1.48 | 1.270 |
| 47.46000 | 9.91 | 1.14 | 0.360 |
| 63.67000 | 3.57 | 10.36 | 0.100 |

| Table Analyzed | LRN FLU |
| --- | --- |
|  |  |
| Friedman test |  |
| P value | < 0,0001 |
| Exact or approximate P value? | Approximate |
| P value summary | **** |
| Are means signif. different? (P < 0.05) | Yes |
| Number of groups | 4 |
| Friedman statistic | 29,54 |
|  |  |
| Data summary |  |
| Number of treatments (columns) | 4 |
| Number of subjects (rows) | 15 |

| Number of families | 1 |  |  |
| --- | --- | --- | --- |
| Number of comparisons per family | 3 |  |  |
| Alpha | 0,05 |  |  |
|  |  |  |  |
| Dunn's multiple comparisons test | Rank sum diff, | Significant? | Summary |
|  |  |  |  |
| Initial test vs. 2nd test | 25,50 | Yes | *** |
| Initial test vs. 3rd test | 28,50 | Yes | *** |
| Initial test vs. 4th test FLU | 36,00 | Yes | **** |

1. **Two-way ANOVA of the 4^th^ test**

| Two-way ANOVA | Ordinary |  |  |  |  |
| --- | --- | --- | --- | --- | --- |
| Alpha | 0,05 |  |  |  |  |
|  |  |  |  |  |  |
| Source of Variation | % of total variation | P value | P value summary | Significant? |  |
| Interaction | 3,031 | 0,1503 | ns | No |  |
| Row Factor | 16,11 | 0,0015 | ** | Yes |  |
| Column Factor | 7,619 | 0,0247 | * | Yes |  |
|  |  |  |  |  |  |
| ANOVA table | SS | DF | MS | F (DFn, DFd) | P value |
| Interaction | 614,6 | 1 | 614,6 | F (1, 51) = 2,133 | P = 0,1503 |
| Row Factor | 3266 | 1 | 3266 | F (1, 51) = 11,33 | P = 0,0015 |
| Column Factor | 1545 | 1 | 1545 | F (1, 51) = 5,360 | P = 0,0247 |
| Residual | 14698 | 51 | 288,2 |  |  |

**Tables 4. Data from Figure 3**

1. **HRN**

| Initial test | 2nd test | 3rd test | 4th test control |
| --- | --- | --- | --- |
| 360.0 | 142.500000 | 19.50 | 129.300 |
| 360.0 | 113.800000 | 36.20 | 21.600 |
| 360.0 | 0.900000 | 0.00 | 326.400 |
| 360.0 | 0.000000 | 15.20 | 15.800 |
| 360.0 | 79.000000 | 0.00 | 104.600 |
| 360.0 | 176.800000 | 84.10 | 0.000 |
| 360.0 | 0.000000 | 347.80 | 0.000 |
| 360.0 | 112.000000 | 0.00 | 0.000 |
| 360.0 | 38.300000 | 0.00 | 0.000 |
| 360.0 | 342.300000 | 4.80 | 3.200 |
| 360.0 | 0.000000 | 154.20 | 57.600 |
| 360.0 | 328.100000 | 48.10 | 287.500 |
| 360.0 | 0.000000 | 201.20 | 0.000 |
| 360.0 | 0.000000 | 136.00 | 134.500 |
| 360.0 | 131.000000 | 4.00 | 0.000 |
| 360.0 | 0.000000 | 177.70 | 0.000 |
| 360.0 | 47.100000 | 19.10 | 0.000 |

| Table Analyzed | HRN CTRL |
| --- | --- |
|  |  |
| Friedman test |  |
| P value | < 0,0001 |
| Exact or approximate P value? | Approximate |
| P value summary | **** |
| Are means signif. different? (P < 0.05) | Yes |
| Number of groups | 4 |
| Friedman statistic | 33,00 |
|  |  |
| Data summary |  |
| Number of treatments (columns) | 4 |
| Number of subjects (rows) | 17 |

| Number of families | 1 |  |  |
| --- | --- | --- | --- |
| Number of comparisons per family | 3 |  |  |
| Alpha | 0,05 |  |  |
|  |  |  |  |
| Dunn's multiple comparisons test | Rank sum diff, | Significant? | Summary |
|  |  |  |  |
| Initial test vs. 2nd test | 29,50 | Yes | *** |
| Initial test vs. 3rd test | 34,00 | Yes | **** |
| Initial test vs. 4th test control | 38,50 | Yes | **** |

Fluoxetine exposed

| Initial test | 2nd test | 3rd test | 4th test FLU |
| --- | --- | --- | --- |
| 360.0 | 154.800000 | 9.70 | 360.000 |
| 360.0 | 0.000000 | 0.00 | 23.100 |
| 360.0 | 153.200000 | 0.00 | 360.000 |
| 360.0 | 0.000000 | 232.70 | 111.800 |
| 360.0 | 118.800000 | 36.70 | 360.000 |
| 360.0 | 195.900000 | 76.80 | 360.000 |
| 360.0 | 0.000000 | 0.00 | 13.600 |
| 360.0 | 0.000000 | 41.80 | 360.000 |
| 360.0 | 63.000000 | 25.00 | 360.000 |
| 360.0 | 0.000000 | 0.00 | 360.000 |
| 360.0 | 0.000000 | 330.70 | 17.300 |

| Table Analyzed | HRN FLU |
| --- | --- |
|  |  |
| Friedman test |  |
| P value | < 0,0001 |
| Exact or approximate P value? | Approximate |
| P value summary | **** |
| Are means signif. different? (P < 0.05) | Yes |
| Number of groups | 4 |
| Friedman statistic | 25,08 |
|  |  |
| Data summary |  |
| Number of treatments (columns) | 4 |
| Number of subjects (rows) | 11 |

| Number of families | 1 |  |  |
| --- | --- | --- | --- |
| Number of comparisons per family | 3 |  |  |
| Alpha | 0,05 |  |  |
|  |  |  |  |
| Dunn's multiple comparisons test | Rank sum diff, | Significant? | Summary |
|  |  |  |  |
| Initial test vs. 2nd test | 23,00 | Yes | *** |
| Initial test vs. 3rd test | 23,00 | Yes | *** |
| Initial test vs. 4th test FLU | 6,000 | No | ns |

**(B) LRN**

| Initial test | 2nd test | 3rd test | 4th test |
| --- | --- | --- | --- |
| 3.10000 | 0.00 | 27.20 | 163.700 |
| 0.00000 | 0.00 | 33.60 | 90.000 |
| 2.80000 | 24.60 | 1.30 | 0.000 |
| 3.70000 | 51.80 | 0.00 | 168.700 |
| 137.40000 | 131.70 | 50.10 | 45.400 |
| 5.70000 | 12.80 | 56.70 | 4.900 |
| 37.70000 | 40.40 | 25.70 | 0.000 |
| 8.40000 | 15.70 | 23.90 | 163.700 |
| 11.90000 | 0.00 | 102.60 | 90.000 |
| 10.10000 | 126.20 | 68.70 | 0.000 |
| 48.40000 | 64.20 | 125.20 | 168.700 |

| Table Analyzed | neophilics CTRL |
| --- | --- |
|  |  |
| Friedman test |  |
| P value | 0,7307 |
| Exact or approximate P value? | Approximate |
| P value summary | ns |
| Are means signif. different? (P < 0.05) | No |
| Number of groups | 4 |
| Friedman statistic | 1,294 |
|  |  |
| Data summary |  |
| Number of treatments (columns) | 4 |
| Number of subjects (rows) | 11 |

**Fluoxetine exposed**

| Initial test | 2nd test | 3rd test | | 4th test FLU |  |
| --- | --- | --- | --- | --- | --- |
| 26.99000 | 10.96 | 1.72 | | 0.100 |  |
| 27.34000 | 13.63 | 5.61 | | 0.100 |  |
| 27.54000 | 23.16 | 10.38 | | 7.150 |  |
| 25.85000 | 5.18 | 0.38 | | 0.100 |  |
| 5.50000 | 189.00 | 2.90 | | 35.100 |  |
| 2.60000 | 54.20 | 118.70 | | 8.200 |  |
| 2.10000 | 83.30 | 15.50 | | 87.600 |  |
| 28.00000 | 0.00 | 6.40 | | 13.600 |  |
| 15.00000 | 0.00 | 185.10 | | 117.600 |  |
| Table Analyzed | | | neophilics FLU | | |
|  | | |  | | |
| Friedman test | | |  | | |
| P value | | | 0,5864 | | |
| Exact or approximate P value? | | | Approximate | | |
| P value summary | | | ns | | |
| Are means signif. different? (P < 0.05) | | | No | | |
| Number of groups | | | 4 | | |
| Friedman statistic | | | 1,933 | | |
|  | | |  | | |
| Data summary | | |  | | |
| Number of treatments (columns) | | | 4 | | |
| Number of subjects (rows) | | | 9 | | |

1. **Two-way ANOVA of the 4^th^** test

| Table Analyzed | Data 2 |  |  |  |  |
| --- | --- | --- | --- | --- | --- |
|  |  |  |  |  |  |
| Two-way ANOVA | Ordinary |  |  |  |  |
| Alpha | 0,05 |  |  |  |  |
|  |  |  |  |  |  |
| Source of Variation | % of total variation | P value | P value summary | Significant? |  |
| Interaction | 18,26 | 0,0013 | ** | Yes |  |
| Row Factor | 12,84 | 0,0062 | ** | Yes |  |
| Column Factor | 5,197 | 0,0740 | ns | No |  |
|  |  |  |  |  |  |
| ANOVA table | SS | DF | MS | F (DFn, DFd) | P value |
| Interaction | 134672 | 1 | 134672 | F (1, 43) = 11,78 | P = 0,0013 |
| Row Factor | 94659 | 1 | 94659 | F (1, 43) = 8,283 | P = 0,0062 |
| Column Factor | 38325 | 1 | 38325 | F (1, 43) = 3,353 | P = 0,0740 |
| Residual | 491424 | 43 | 11428 |  |  |

| Compare cell means regardless of rows and columns |  |  |  |  |
| --- | --- | --- | --- | --- |
|  |  |  |  |  |
| Number of families | 1 |  |  |  |
| Number of comparisons per family | 6 |  |  |  |
| Alpha | 0,05 |  |  |  |
|  |  |  |  |  |
| Tukey's multiple comparisons test | Mean Diff, | 95% CI of diff, | Significant? | Summary |
|  |  |  |  |  |
| HRN:FLU - vs. HRN:FLU + | -169,0 | -282,9 to -55,17 | Yes | ** |
| HRN:FLU - vs. LRN:FLU - | -17,81 | -128,4 to 92,74 | No | ns |
| HRN:FLU - vs. LRN:FLU + | 33,61 | -84,16 to 151,4 | No | ns |
| HRN:FLU + vs. LRN:FLU - | 151,2 | 26,38 to 276,0 | Yes | * |
| HRN:FLU + vs. LRN:FLU + | 202,6 | 71,36 to 333,9 | Yes | *** |
| LRN:FLU - vs. LRN:FLU + | 51,42 | -76,99 to 179,8 | No | ns |

**Table 5. Data from Figure 4A. Time spent in the novel segment object of non-classified zebrafish population.**

| Control | FLU |
| --- | --- |
| 30.2 | 0.0 |
| 0.8 | 46.5 |
| 0.2 | 74.2 |
| 1.8 | 0.0 |
| 5.6 | 2.2 |
| 0.5 | 115.5 |
| 9.1 | 1.1 |
| 101.4 | 75.9 |
| 128.8 | 0.8 |
| 4.8 | 42.5 |
| 0.4 | 2.3 |
| 8.8 | 10.4 |

| Unpaired t test |  |
| --- | --- |
| P value | 0,7020 |
| P value summary | ns |
| Significantly different? (P < 0.05) | No |
| One- or two-tailed P value? | Two-tailed |
| t, df | t=0,3876 df=22 |

**Table 6. Data from Figure 4B. Latency to the first entry in the zone with the novel object of non-classified zebrafish population.**

| Control | FLU |
| --- | --- |
| 13.7 | 0.0 |
| 351.0 | 0.0 |
| 323.2 | 20.5 |
| 106.2 | 0.0 |
| 131.7 | 205.6 |
| 342.9 | 2.2 |
| 9.8 | 1.2 |
| 28.0 | 0.0 |
| 1.5 | 21.0 |
| 101.6 | 34.2 |
| 67.3 | 153.5 |
| 4.8 | 8.2 |

| Mann Whitney test |  |
| --- | --- |
| P value | 0,0269 |
| Exact or approximate P value? | Exact |
| P value summary | * |
| Significantly different? (P < 0.05) | Yes |
| One- or two-tailed P value? | Two-tailed |
| Sum of ranks in column A,B | 188,0 , 112,0 |
| Mann-Whitney U | 34,00 |

**Table 7. Data from Figure 5. Whole-body cortisol levels of zebrafish during the 43-day period of housing in the individualization structure.**

| Pre | Day 0 | Day 20 | Day 28 | Day 43 |
| --- | --- | --- | --- | --- |
| 5.0 | 7.0 | 14.0 | 3.0 | 13.5 |
| 4.0 | 28.0 | 16.0 | 13.0 | 5.0 |
| 4.0 | 6.0 | 37.0 | 12.5 | 22.0 |
| 2.0 | 5.5 | 26.0 | 6.0 | 7.5 |
| 4.0 | 11.0 | 7.5 | 50.0 | 6.0 |
| 4.0 | 44.0 | 13.0 | 30.0 | 8.0 |
| 5.0 | 41.0 | 7.5 | 6.0 | 30.5 |
|  | 66.5 |  | 26.5 | 13.5 |

| Kruskal-Wallis test |  |
| --- | --- |
| P value | 0,0038 |
| Exact or approximate P value? | Approximate |
| P value summary | ** |
| Do the medians vary signif. (P < 0.05) | Yes |
| Number of groups | 5 |
| Kruskal-Wallis statistic | 15,51 |

| Dunn's multiple comparisons test | Mean rank diff, | Significant? | Summary |
| --- | --- | --- | --- |
|  |  |  |  |
| Pre vs. Day 0 | -19,69 | Yes | ** |
| Pre vs. Day 20 | -19,64 | Yes | ** |
| Pre vs. Day 28 | -16,19 | Yes | * |
| Pre vs. Day 43 | -15,81 | No | ns |
| Day 0 vs. Day 20 | 0,04464 | No | ns |
| Day 0 vs. Day 28 | 3,500 | No | ns |
| Day 0 vs. Day 43 | 3,875 | No | ns |
| Day 20 vs. Day 28 | 3,455 | No | ns |
| Day 20 vs. Day 43 | 3,830 | No | ns |
| Day 28 vs. Day 43 | 0,3750 | No | ns |

**Table 8. Time spent in the new object zone of fish classified as HRN and LRN according the color of the new object and comparison between both colors. (data of the figure 6 A).**

|  | HRN blue | HRN green | LRN blue | LRN green |
| --- | --- | --- | --- | --- |
|  | 0.0 | 1.3 | 55.4 | 83.7 |
|  | 0.2 | 1.7 | 73.2 | 55.6 |
|  | 0.3 | 1.1 | 49.4 | 79.2 |
|  | 0.0 | 0.0 | 66.4 | 103.9 |
|  | 0.0 | 0.2 | 80.4 | 52.7 |
|  | 1.1 | 0.0 | 50.9 | 117.6 |
|  | 0.2 | 0.0 | 59.5 | 85.6 |
|  | 0.0 | 0.0 | 69.2 | 127.7 |
|  | 0.0 | 0.7 | 165.7 | 72.6 |
|  | 0.0 | 0.0 | 76 |  |
|  |  |  | 129.7 |  |
| Mean | 0.18 | 0.5 | 74.16 | 85.51 |
| SEM | 0.1084 | 0.2062 | 10.15 | 8.59 |
| Student’s t test | P = 0.1861, t = 1.375, DF = 18 | | P = 0.6364, t = 0.4809, DF = 18 | |

**Table 9. Data from Figure 6. Whole-body cortisol levels of zebrafish classified as HRN and LRN in response to an acute stress challenge. (ng/g tissue).**

| control | | stressed | |
| --- | --- | --- | --- |
| LRN | HRN | LRN | HRN |
| 1.8 | 4 | 6 | 12.9 |
| 1.4 | 0.8 | 3.6 | 2.8 |
| 1.4 | 2.3 | 4.1 | 5.4 |
| 1.2 | 4.6 | 8.1 | 11.4 |
| 3.9 | 3.6 | 6 | 0.9 |
| 1.2 | 1.8 | 7.4 | 1.8 |
| 3 | 0.7 | 9 | 3.9 |
| 1.8 | 0.7 | 4.1 | 3.8 |
| 3.6 | 3.8 | 1.8 | 0.9 |
| 5.4 | 3.8 | 13 | 1.8 |

| Table Analyzed | Data 2 |  |  |  |  |
| --- | --- | --- | --- | --- | --- |
|  |  |  |  |  |  |
| Two-way ANOVA | Ordinary |  |  |  |  |
| Alpha | 0,05 |  |  |  |  |
|  |  |  |  |  |  |
| Source of Variation | % of total variation | P value | P value summary | Significant? |  |
| Interaction | 2,255 | 0,3050 | ns | No |  |
| Row Factor | 1,636 | 0,3812 | ns | No |  |
| Column Factor | 21,16 | 0,0030 | ** | Yes |  |
|  |  |  |  |  |  |
| ANOVA table | SS | DF | MS | F (DFn, DFd) | P value |
| Interaction | 8,930 | 1 | 8,930 | F (1, 36) = 1,083 | P = 0,3050 |
| Row Factor | 6,480 | 1 | 6,480 | F (1, 36) = 0,7859 | P = 0,3812 |
| Column Factor | 83,81 | 1 | 83,81 | F (1, 36) = 10,16 | P = 0,0030 |
| Residual | 296,8 | 36 | 8,246 |  |  |

| Compare cell means regardless of rows and columns |  |  |  |  |  |  |  |  |
| --- | --- | --- | --- | --- | --- | --- | --- | --- |
|  |  |  |  |  |  |  |  |  |
| Number of families | 1 |  |  |  |  |  |  |  |
| Number of comparisons per family | 6 |  |  |  |  |  |  |  |
| Alpha | 0,05 |  |  |  |  |  |  |  |
|  |  |  |  |  |  |  |  |  |
| Tukey's multiple comparisons test | Mean Diff, | 95% CI of diff, | Significant? | Summary |  |  |  |  |
|  |  |  |  |  |  |  |  |  |
| HRN:S- vs. HRN:S+ | -1,950 | -5,409 to 1,509 | No | ns |  |  |  |  |
| HRN:S- vs. LRN:S- | 0,1400 | -3,319 to 3,599 | No | ns |  |  |  |  |
| HRN:S- vs. LRN:S+ | -3,700 | -7,159 to -0,2414 | Yes | * |  |  |  |  |
| HRN:S+ vs. LRN:S- | 2,090 | -1,369 to 5,549 | No | ns |  |  |  |  |
| HRN:S+ vs. LRN:S+ | -1,750 | -5,209 to 1,709 | No | ns |  |  |  |  |
| LRN:S- vs. LRN:S+ | -3,840 | -7,299 to -0,3814 | Yes | * |  |  |  |  |
|  |  |  |  |  |  |  |  |  |
|  |  |  |  |  |  |  |  |  |
| Test details | Mean 1 | Mean 2 | Mean Diff, | SE of diff, | N1 | N2 | q | DF |
|  |  |  |  |  |  |  |  |  |
| HRN:S- vs. HRN:S+ | 2,610 | 4,560 | -1,950 | 1,284 | 10 | 10 | 2,147 | 36 |
| HRN:S- vs. LRN:S- | 2,610 | 2,470 | 0,1400 | 1,284 | 10 | 10 | 0,1542 | 36 |
| HRN:S- vs. LRN:S+ | 2,610 | 6,310 | -3,700 | 1,284 | 10 | 10 | 4,075 | 36 |
| HRN:S+ vs. LRN:S- | 4,560 | 2,470 | 2,090 | 1,284 | 10 | 10 | 2,302 | 36 |
| HRN:S+ vs. LRN:S+ | 4,560 | 6,310 | -1,750 | 1,284 | 10 | 10 | 1,927 | 36 |
| LRN:S- vs. LRN:S+ | 2,470 | 6,310 | -3,840 | 1,284 | 10 | 10 | 4,229 | 36 |

Table 10. Locomotor parameters in zebrafish classified according their responsivity to novelty. HRN = high responders to novelty, LRN = low responders to novelty, FLU = fluoxetine.

|  |  | 1^st^ test | 2^nd^ test | 3^rd^ test | 4^th^ test | *P* value | *Statistics of two-way ANOVA of 4^th^ test* |
| --- | --- | --- | --- | --- | --- | --- | --- |
| Total distance (m) | HRN control | 12.74 ± 1.63 | 6.57 ± 1.23* | 7.99 ± 1.48 | 11.33 ± 1.44 | 0.0087 | Interaction  F_1,47_=2.689 , *p*=0.1077  Personality  F_1,47_=0.2040 , *p*=0.6536  FLU exposure  F_1,47_=2.565, *p*=0.1159 |
|  | HRN FLU | 15.01 ± 1.95 | 9.08 ± 0.92* | 10.28 ± 1.01 | 11.43 ± 1.13 | 0.0315 |  |
|  | LRN control | 9.08 ± 0.84 | 8.37 ± 0.81 | 3.91 ± 0.83* | 16.61 ± 5.72 | 0.0277 |  |
|  | LRN FLU | 8.36 ± 1.79 | 5.74 ± 0.68 | 6.78 ± 1.02 | 8.81 ± 0.95 | 0.0832 |  |
| Line crossings (unit) | HRN control | 183 ± 26 | 129 ± 17 | 138 ± 21 | 180 ± 27 | 0.1167 | Interaction  F_1,47_=0.026 , *p*=0.8713  Personality  F_1,47_=3.565 , *p*=0.0665  FLU exposure  F_1,47_=0.054, *p*=0.8162 |
|  | HRN FLU | 237 ± 28 | 129 ± 17* | 153 ± 17 | 181 ± 17 | 0.0121 |  |
|  | LRN control | 165 ± 21 | 132 ± 25 | 68 ± 16 | 140 ± 21 | 0.0681 |  |
|  | LRN FLU | 124 ± 17 | 104 ± 13 | 99 ± 17 | 132 ± 18 | 0.1059 |  |
| Absolute turn angle (^o^) | HRN control | 53005 ± 6714 | 35444 ± 2427 | 30409 ± 3291* | 46465 ± 3860 | 0.0025 | Interaction  F_1,47_=0.3005 , *p*=0.5863  Personality  F_1,47_=1.885 , *p*=0.1766  FLU exposure  F_1,47_=3.694, *p*=0.0610 |
|  | HRN FLU | 55962 ± 4414 | 34620 ± 2621* | 34457 ± 1978* | 38481 ± 2196* | 0.0002 |  |
|  | LRN control | 58036 ± 4585 | 45994 ± 1371 | 19702 ± 3900* | 40507 ± 3422 | 0.0004 |  |
|  | LRN FLU | 47536 ± 5828 | 31389 ± 2824* | 32908 ± 2132* | 35556 ± 1922 | 0.0004 |  |
| Rotations (units) | HRN control | 22 ± 5 | 15 ± 2 | 11 ± 2 | 23 ± 4 | 0.0098 | Interaction  F_1,47_=1.058 , *p*=0.3098  Personality  F_1,47_=1.312 , *p*=0.2588  FLU exposure  F_1,47_=3.554, *p*=0.0667 |
|  | HRN FLU | 33 ± 5 | 22 ± 2 | 22 ± 3 | 28 ± 4 | 0.0600 |  |
|  | LRN control | 18 ± 3 | 12 ± 2 | 5 ± 1* | 17 ± 3 | 0.0010 |  |
|  | LRN FLU | 23 ± 3 | 17 ± 3 | 20 ± 4 | 26 ± 4 | 0.0501 |  |

The asterisks indicate statistical differences between the four tests within the same group (differences in line) compared by a repeated measures ANOVA, *p values are depicted in the table.

Repeated measures ANOVA (line comparisons)

Raw data and statistics of Table 9.

Distance

LRN control

| Initial test | 2nd test | 3rd test | 4th test control |
| --- | --- | --- | --- |
| 5.447000 | 13.592000 | 12.191 | 13.544 |
| 11.358000 | 6.682000 | 13.222 | 0.000 |
| 6.586000 | 2.344000 | 11.755 | 12.203 |
| 20.932000 | 12.651000 | 12.875 | 14.606 |
| 11.956000 | 12.883000 | 15.085 | 15.945 |
| 13.521000 | 11.717000 | 12.972 | 17.626 |
| 13.741000 | 7.553000 | 9.915 | 9.403 |
| 6.818000 | 9.875000 | 14.639 | 14.520 |
| 27.274000 | 12.242000 | 11.523 | 15.635 |
| 12.036000 | 11.870000 | 10.854 | 9.085 |
| 24.179000 | 2.850000 | 0.291 | 7.584 |
| 10.033000 | 9.741000 | 7.833 | 8.839 |
| 30.596000 | 5.914000 | 6.912 | 8.838 |
| 15.321000 | 7.798000 | 5.461 | 10.802 |
| 15.321000 | 8.446000 | 8.720 | 12.807 |

| Table Analyzed | LRN CTRL |
| --- | --- |
|  |  |
| Friedman test |  |
| P value | 0,0315 |
| Exact or approximate P value? | Approximate |
| P value summary | * |
| Are means signif. different? (P < 0.05) | Yes |
| Number of groups | 4 |
| Friedman statistic | 8,840 |
|  |  |
| Data summary |  |
| Number of treatments (columns) | 4 |
| Number of subjects (rows) | 15 |

| Number of families | 1 |  |  |  |  |
| --- | --- | --- | --- | --- | --- |
| Number of comparisons per family | 3 |  |  |  |  |
| Alpha | 0,05 |  |  |  |  |
|  |  |  |  |  |  |
| Dunn's multiple comparisons test | Rank sum diff, | Significant? | Summary |  |  |
|  |  |  |  |  |  |
| Initial test vs. 2nd test | 19,00 | Yes | * |  |  |
| Initial test vs. 3rd test | 14,00 | No | ns |  |  |
| Initial test vs. 4th test control | 5,000 | No | ns |  |  |
|  |  |  |  |  |  |
|  |  |  |  |  |  |
| Test details | Rank sum 1 | Rank sum 2 | Rank sum diff, | n1 | n2 |
|  |  |  |  |  |  |
| Initial test vs. 2nd test | 47,00 | 28,00 | 19,00 | 15 | 15 |
| Initial test vs. 3rd test | 47,00 | 33,00 | 14,00 | 15 | 15 |
| Initial test vs. 4th test control | 47,00 | 42,00 | 5,000 | 15 | 15 |

LRN FLU

| Initial test | 2nd test | 3rd test | 4th test FLU |
| --- | --- | --- | --- |
| 17.314 | 13.818000 | 12.470 | 13.778 |
| 12.870 | 9.773000 | 11.005 | 13.106 |
| 2.850 | 5.484000 | 15.371 | 8.046 |
| 22.729 | 8.837000 | 9.149 | 18.278 |
| 14.547 | 7.355000 | 2.014 | 17.818 |
| 9.077 | 6.718000 | 10.243 | 8.373 |
| 11.734 | 2.189000 | 5.620 | 9.628 |
| 11.972 | 7.786000 | 1.561 | 5.518 |
| 11.304 | 1.070000 | 3.187 | 5.843 |
| 13.023 | 2.669000 | 9.379 | 12.943 |

| Table Analyzed | LRN FLU |
| --- | --- |
|  |  |
| Friedman test |  |
| P value | 0,0087 |
| Exact or approximate P value? | Approximate |
| P value summary | ** |
| Are means signif. different? (P < 0.05) | Yes |
| Number of groups | 4 |
| Friedman statistic | 11,64 |
|  |  |
| Data summary |  |
| Number of treatments (columns) | 4 |
| Number of subjects (rows) | 10 |

| Number of families | 1 |  |  |  |  |
| --- | --- | --- | --- | --- | --- |
| Number of comparisons per family | 3 |  |  |  |  |
| Alpha | 0,05 |  |  |  |  |
|  |  |  |  |  |  |
| Dunn's multiple comparisons test | Rank sum diff, | Significant? | Summary |  |  |
|  |  |  |  |  |  |
| Initial test vs. 2nd test | 18,00 | Yes | ** |  |  |
| Initial test vs. 3rd test | 13,00 | No | ns |  |  |
| Initial test vs. 4th test FLU | 5,000 | No | ns |  |  |
|  |  |  |  |  |  |
|  |  |  |  |  |  |
| Test details | Rank sum 1 | Rank sum 2 | Rank sum diff, | n1 | n2 |
|  |  |  |  |  |  |
| Initial test vs. 2nd test | 34,00 | 16,00 | 18,00 | 10 | 10 |
| Initial test vs. 3rd test | 34,00 | 21,00 | 13,00 | 10 | 10 |
| Initial test vs. 4th test FLU | 34,00 | 29,00 | 5,000 | 10 | 10 |

HRN control

| Initial test | 2nd test | 3rd test | 4th test |
| --- | --- | --- | --- |
| 38.93333 | 4.900 | 2.900 | 2.800 |
| 7.83500 | 7.607 | 10.489 | 13.259 |
| 4.62800 | 0.156 | 7.143 | 9.161 |
| 14.33000 | 7.468 | 10.387 | 10.810 |
| 4.40500 | 4.367 | 2.826 | 4.052 |
| 0.56100 | 8.786 | 12.356 | 9.905 |
| 9.18900 | 8.350 | 2.757 | 1.596 |
| 7.14800 | 6.182 | 6.018 | 10.697 |
| 4.46100 | 5.327 | 7.486 | 5.044 |
| 6.61500 | 3.868 | 9.688 | 9.384 |
| 29.24400 | 1.753 | 1.482 | 7.050 |
| 10.83200 | 9.174 | 6.633 | 15.481 |
| 6.36900 | 4.479 | 5.035 | 10.229 |
| 1.23200 | 4.979 | 5.262 | 11.373 |
| 13.18100 | 8.457 | 13.635 | 9.291 |
| 5.30900 | 5.194 | 0.552 | 4.851 |

| Table Analyzed | HRN CTRL |
| --- | --- |
|  |  |
| Friedman test |  |
| P value | 0,1401 |
| Exact or approximate P value? | Approximate |
| P value summary | ns |
| Are means signif. different? (P < 0.05) | No |
| Number of groups | 4 |
| Friedman statistic | 5,475 |
|  |  |
| Data summary |  |
| Number of treatments (columns) | 4 |
| Number of subjects (rows) | 16 |

HRN FLU

| Initial test | 2nd test | 3rd test | 4th test FLU |
| --- | --- | --- | --- |
| 13.84200 | 11.365 | 2.327 | 5.711 |
| 9.36400 | 12.123 | 0.869 | 24.300 |
| 10.17400 | 5.302 | 0.298 | 12.975 |
| 4.96800 | 7.878 | 2.186 | 64.990 |
| 11.42000 | 8.459 | 5.006 | 4.182 |
| 11.04000 | 9.651 | 4.366 | 16.500 |
| 7.27000 | 4.392 | 5.451 | 4.342 |
| 8.87900 | 6.638 | 5.720 | 8.822 |
| 7.56600 | 10.488 | 3.811 | 13.024 |
| 6.30300 | 7.381 | 9.093 | 11.168 |

| Table Analyzed | HRN FLU |
| --- | --- |
|  |  |
| Friedman test |  |
| P value | 0,0277 |
| Exact or approximate P value? | Approximate |
| P value summary | * |
| Are means signif. different? (P < 0.05) | Yes |
| Number of groups | 4 |
| Friedman statistic | 9,120 |
|  |  |
| Data summary |  |
| Number of treatments (columns) | 4 |
| Number of subjects (rows) | 10 |

| Number of families | 1 |  |  |  |  |
| --- | --- | --- | --- | --- | --- |
| Number of comparisons per family | 3 |  |  |  |  |
| Alpha | 0,05 |  |  |  |  |
|  |  |  |  |  |  |
| Dunn's multiple comparisons test | Rank sum diff, | Significant? | Summary |  |  |
|  |  |  |  |  |  |
| Initial test vs. 2nd test | 4,000 | No | ns |  |  |
| Initial test vs. 3rd test | 14,00 | Yes | * |  |  |
| Initial test vs. 4th test FLU | -2,000 | No | ns |  |  |
|  |  |  |  |  |  |
|  |  |  |  |  |  |
| Test details | Rank sum 1 | Rank sum 2 | Rank sum diff, | n1 | n2 |
|  |  |  |  |  |  |
| Initial test vs. 2nd test | 29,00 | 25,00 | 4,000 | 10 | 10 |
| Initial test vs. 3rd test | 29,00 | 15,00 | 14,00 | 10 | 10 |
| Initial test vs. 4th test FLU | 29,00 | 31,00 | -2,000 | 10 | 10 |

Two-way ANOVA of 4^th^ test

| Table Analyzed | distance |  |  |  |  |
| --- | --- | --- | --- | --- | --- |
|  |  |  |  |  |  |
| Two-way ANOVA | Ordinary |  |  |  |  |
| Alpha | 0,05 |  |  |  |  |
|  |  |  |  |  |  |
| Source of Variation | % of total variation | P value | P value summary | Significant? |  |
| Interaction | 5,138 | 0,1077 | ns | No |  |
| Row Factor | 0,3899 | 0,6536 | ns | No |  |
| Column Factor | 4,902 | 0,1159 | ns | No |  |
|  |  |  |  |  |  |
| ANOVA table | SS | DF | MS | F (DFn, DFd) | P value |
| Interaction | 207,3 | 1 | 207,3 | F (1, 47) = 2,689 | P = 0,1077 |
| Row Factor | 15,73 | 1 | 15,73 | F (1, 47) = 0,2040 | P = 0,6536 |
| Column Factor | 197,8 | 1 | 197,8 | F (1, 47) = 2,565 | P = 0,1159 |
| Residual | 3624 | 47 | 77,11 |  |  |
|  |  |  |  |  |  |

Crossings

LRN control

| Initial test | 2nd test | 3rd test | 4th test control |
| --- | --- | --- | --- |
| 183.0 | 29.000000 | 191.00 | 173.000 |
| 253.0 | 203.000000 | 221.00 | 218.000 |
| 139.0 | 213.000000 | 215.00 | 250.000 |
| 206.0 | 194.000000 | 205.00 | 281.000 |
| 265.0 | 109.000000 | 129.00 | 89.000 |
| 68.0 | 165.000000 | 169.00 | 201.000 |
| 386.0 | 140.000000 | 180.00 | 243.000 |
| 149.0 | 177.000000 | 176.00 | 122.000 |
| 408.0 | 21.000000 | 5.00 | 125.000 |
| 165.0 | 119.000000 | 120.00 | 138.000 |
| 368.0 | 134.000000 | 142.00 | 184.000 |
| 250.0 | 103.000000 | 82.00 | 106.000 |
| 250.0 | 79.000000 | 150.00 | 228.000 |

| Table Analyzed | LRN CTRL |
| --- | --- |
|  |  |
| Friedman test |  |
| P value | 0,0121 |
| Exact or approximate P value? | Approximate |
| P value summary | * |
| Are means signif. different? (P < 0.05) | Yes |
| Number of groups | 4 |
| Friedman statistic | 10,94 |
|  |  |
| Data summary |  |
| Number of treatments (columns) | 4 |
| Number of subjects (rows) | 13 |

| Number of families | 1 |  |  |  |  |
| --- | --- | --- | --- | --- | --- |
| Number of comparisons per family | 3 |  |  |  |  |
| Alpha | 0,05 |  |  |  |  |
|  |  |  |  |  |  |
| Dunn's multiple comparisons test | Rank sum diff, | Significant? | Summary |  |  |
|  |  |  |  |  |  |
| Initial test vs. 2nd test | 21,00 | Yes | ** |  |  |
| Initial test vs. 3rd test | 11,00 | No | ns |  |  |
| Initial test vs. 4th test control | 6,000 | No | ns |  |  |
|  |  |  |  |  |  |
|  |  |  |  |  |  |
| Test details | Rank sum 1 | Rank sum 2 | Rank sum diff, | n1 | n2 |
|  |  |  |  |  |  |
| Initial test vs. 2nd test | 42,00 | 21,00 | 21,00 | 13 | 13 |
| Initial test vs. 3rd test | 42,00 | 31,00 | 11,00 | 13 | 13 |
| Initial test vs. 4th test control | 42,00 | 36,00 | 6,000 | 13 | 13 |

LRN FLU

| Initial test | 2nd test | 3rd test | 4th test FLU |
| --- | --- | --- | --- |
| 104.000 | 165.000000 | 211.000 | 166.000 |
| 258.000 | 106.000000 | 187.000 | 65.000 |
| 238.000 | 233.000000 | 215.000 | 228.000 |
| 171.000 | 45.000000 | 162.000 | 185.000 |
| 37.000 | 82.000000 | 185.000 | 128.000 |
| 330.000 | 145.000000 | 127.000 | 246.000 |
| 224.000 | 178.000000 | 24.000 | 352.000 |
| 138.000 | 123.000000 | 132.000 | 151.000 |
| 144.000 | 93.000000 | 77.000 | 204.000 |
| 189.000 | 127.000000 | 62.000 | 75.000 |

| Table Analyzed | LRN FLU |
| --- | --- |
|  |  |
| Friedman test |  |
| P value | 0,1116 |
| Exact or approximate P value? | Approximate |
| P value summary | ns |
| Are means signif. different? (P < 0.05) | No |
| Number of groups | 4 |
| Friedman statistic | 6,000 |
|  |  |
| Data summary |  |
| Number of treatments (columns) | 4 |
| Number of subjects (rows) | 10 |

HRN control

| Initial test | 2nd test | 3rd test | 4th test |
| --- | --- | --- | --- |
| 17.80000 | 3.20 | 47.30 | 108.900 |
| 38.93333 | 4.90 | 2.90 | 2.800 |
| 161.00000 | 110.00 | 144.00 | 197.000 |
| 80.00000 | 2.00 | 16.00 | 125.000 |
| 188.00000 | 117.00 | 165.00 | 195.000 |
| 117.00000 | 47.00 | 90.00 | 38.000 |
| 18.00000 | 122.00 | 192.00 | 158.000 |
| 155.00000 | 142.00 | 21.00 | 6.000 |
| 46.00000 | 134.00 | 114.00 | 182.000 |
| 144.00000 | 72.00 | 169.00 | 128.000 |
| 175.00000 | 145.00 | 50.00 | 174.000 |
| 132.00000 | 124.00 | 64.00 | 176.000 |
| 86.00000 | 71.00 | 69.00 | 182.000 |
| 228.00000 | 157.00 | 170.00 | 89.000 |
| 93.00000 | 103.00 | 22.00 | 58.000 |

| Table Analyzed | HRN CTRL |
| --- | --- |
|  |  |
| Friedman test |  |
| P value | 0,1059 |
| Exact or approximate P value? | Approximate |
| P value summary | ns |
| Are means signif. different? (P < 0.05) | No |
| Number of groups | 4 |
| Friedman statistic | 6,120 |
|  |  |
| Data summary |  |
| Number of treatments (columns) | 4 |
| Number of subjects (rows) | 15 |

HRN FLU

| Initial test | 2nd test | 3rd test | 4th test FLU |
| --- | --- | --- | --- |
| 214.00000 | 162.000 | 34.000 | 97.000 |
| 141.00000 | 245.000 | 8.000 | 186.000 |
| 194.00000 | 52.000 | 9.000 | 163.000 |
| 95.00000 | 214.000 | 28.000 | 60.000 |
| 199.00000 | 64.000 | 129.000 | 65.000 |
| 170.00000 | 170.000 | 58.000 | 205.000 |
| 303.00000 | 103.000 | 109.000 | 89.000 |
| 149.00000 | 4.000 | 70.000 | 99.000 |
| 60.00000 | 201.000 | 44.000 | 259.000 |
| 134.00000 | 111.000 | 158.000 | 177.000 |

| Table Analyzed | HRN FLU |
| --- | --- |
|  |  |
| Friedman test |  |
| P value | 0,0681 |
| Exact or approximate P value? | Approximate |
| P value summary | ns |
| Are means signif. different? (P < 0.05) | No |
| Number of groups | 4 |
| Friedman statistic | 7,121 |
|  |  |
| Data summary |  |
| Number of treatments (columns) | 4 |
| Number of subjects (rows) | 10 |

Two-way ANOVA of 4^th^ test

| Table Analyzed | crossings |  |  |  |  |
| --- | --- | --- | --- | --- | --- |
|  |  |  |  |  |  |
| Two-way ANOVA | Ordinary |  |  |  |  |
| Alpha | 0,05 |  |  |  |  |
|  |  |  |  |  |  |
| Source of Variation | % of total variation | P value | P value summary | Significant? |  |
| Interaction | 0,05951 | 0,8713 | ns | No |  |
| Row Factor | 7,987 | 0,0661 | ns | No |  |
| Column Factor | 0,1227 | 0,8162 | ns | No |  |
|  |  |  |  |  |  |
| ANOVA table | SS | DF | MS | F (DFn, DFd) | P value |
| Interaction | 112,2 | 1 | 112,2 | F (1, 47) = 0,02657 | P = 0,8713 |
| Row Factor | 15054 | 1 | 15054 | F (1, 47) = 3,565 | P = 0,0661 |
| Column Factor | 231,2 | 1 | 231,2 | F (1, 47) = 0,05475 | P = 0,8162 |
| Residual | 173121 | 47 | 4222 |  |  |

Rotations

LRN control

| Initial test | 2nd test | 3rd test | 4th test control |
| --- | --- | --- | --- |
| 6.0 | 25.000000 | 21.00 | 26.000 |
| 28.0 | 30.000000 | 27.00 | 0.000 |
| 22.0 | 11.000000 | 37.00 | 35.000 |
| 44.0 | 33.000000 | 27.00 | 37.000 |
| 15.0 | 20.000000 | 34.00 | 40.000 |
| 31.0 | 14.000000 | 26.00 | 35.000 |
| 35.0 | 25.000000 | 20.00 | 31.000 |
| 14.0 | 28.000000 | 43.00 | 41.000 |
| 42.0 | 32.000000 | 15.00 | 40.000 |
| 38.0 | 22.000000 | 22.00 | 18.000 |
| 57.0 | 13.000000 | 0.00 | 19.000 |
| 27.0 | 13.000000 | 7.00 | 11.000 |
| 77.0 | 16.000000 | 16.00 | 24.000 |
| 26.0 | 17.000000 | 17.00 | 31.000 |
| 26.0 | 34.000000 | 14.00 | 28.000 |

| Table Analyzed | LRN CTRL |
| --- | --- |
|  |  |
| Friedman test |  |
| P value | 0,0600 |
| Exact or approximate P value? | Approximate |
| P value summary | ns |
| Are means signif. different? (P < 0.05) | No |
| Number of groups | 4 |
| Friedman statistic | 7,408 |
|  |  |
| Data summary |  |
| Number of treatments (columns) | 4 |
| Number of subjects (rows) | 15 |

LRN FLU

| Initial test | 2nd test | 3rd test | 4th test FLU |
| --- | --- | --- | --- |
| 30.000 | 11.000000 | 17.000 | 48.000 |
| 6.000 | 12.000000 | 28.000 | 16.000 |
| 20.000 | 9.000000 | 10.000 | 12.000 |
| 30.000 | 20.000000 | 7.000 | 32.000 |
| 6.000 | 9.000000 | 9.000 | 11.000 |
| 54.000 | 28.000000 | 7.000 | 24.000 |
| 16.000 | 15.000000 | 4.000 | 34.000 |
| 12.000 | 13.000000 | 12.000 | 14.000 |
| 18.000 | 18.000000 | 12.000 | 21.000 |
| 31.000 | 14.000000 | 10.000 | 21.000 |

| Table Analyzed | LRN FLU |
| --- | --- |
|  |  |
| Friedman test |  |
| P value | 0,0098 |
| Exact or approximate P value? | Approximate |
| P value summary | ** |
| Are means signif. different? (P < 0.05) | Yes |
| Number of groups | 4 |
| Friedman statistic | 11,38 |
|  |  |
| Data summary |  |
| Number of treatments (columns) | 4 |
| Number of subjects (rows) | 10 |

| Number of families | 1 |  |  |  |  |
| --- | --- | --- | --- | --- | --- |
| Number of comparisons per family | 3 |  |  |  |  |
| Alpha | 0,05 |  |  |  |  |
|  |  |  |  |  |  |
| Dunn's multiple comparisons test | Rank sum diff, | Significant? | Summary |  |  |
|  |  |  |  |  |  |
| Initial test vs. 2nd test | 6,000 | No | ns |  |  |
| Initial test vs. 3rd test | 10,00 | No | ns |  |  |
| Initial test vs. 4th test FLU | -8,000 | No | ns |  |  |
|  |  |  |  |  |  |
|  |  |  |  |  |  |
| Test details | Rank sum 1 | Rank sum 2 | Rank sum diff, | n1 | n2 |
|  |  |  |  |  |  |
| Initial test vs. 2nd test | 27,00 | 21,00 | 6,000 | 10 | 10 |
| Initial test vs. 3rd test | 27,00 | 17,00 | 10,00 | 10 | 10 |
| Initial test vs. 4th test FLU | 27,00 | 35,00 | -8,000 | 10 | 10 |

HRN Control

| Initial test | 2nd test | 3rd test | 4th test |
| --- | --- | --- | --- |
| 25.00000 | 16.00 | 23.00 | 20.000 |
| 13.00000 | 0.00 | 40.00 | 34.000 |
| 27.00000 | 20.00 | 28.00 | 16.000 |
| 10.00000 | 3.00 | 11.00 | 16.000 |
| 2.00000 | 30.00 | 38.00 | 44.000 |
| 32.00000 | 26.00 | 2.00 | 2.000 |
| 28.00000 | 19.00 | 28.00 | 32.000 |
| 20.00000 | 18.00 | 18.00 | 11.000 |
| 15.00000 | 12.00 | 18.00 | 32.000 |
| 52.00000 | 4.00 | 9.00 | 38.000 |
| 39.00000 | 37.00 | 26.00 | 42.000 |
| 29.00000 | 13.00 | 10.00 | 35.000 |
| 3.00000 | 20.00 | 21.00 | 32.000 |
| 30.00000 | 30.00 | 34.00 | 26.000 |
| 21.00000 | 12.00 | 2.00 | 15.000 |

| Table Analyzed | HRN CTRL |
| --- | --- |
|  |  |
| Friedman test |  |
| P value | 0,0506 |
| Exact or approximate P value? | Approximate |
| P value summary | ns |
| Are means signif. different? (P < 0.05) | No |
| Number of groups | 4 |
| Friedman statistic | 7,788 |
|  |  |
| Data summary |  |
| Number of treatments (columns) | 4 |
| Number of subjects (rows) | 15 |

HRN FLU

| Initial test | 2nd test | 3rd test | 4th test FLU |
| --- | --- | --- | --- |
| 5.00000 | 10.000 | 4.000 | 14.000 |
| 19.00000 | 9.000 | 0.000 | 17.000 |
| 16.00000 | 8.000 | 0.000 | 23.000 |
| 22.00000 | 19.000 | 4.000 | 14.000 |
| 38.00000 | 22.000 | 8.000 | 14.000 |
| 19.00000 | 12.000 | 2.000 | 33.000 |
| 17.00000 | 9.000 | 3.000 | 5.000 |
| 22.00000 | 13.000 | 6.000 | 8.000 |
| 15.00000 | 9.000 | 5.000 | 23.000 |
| 12.00000 | 11.000 | 17.000 | 20.000 |
|  |  |  |  |

| Table Analyzed | HRN FLU |
| --- | --- |
|  |  |
| Friedman test |  |
| P value | 0,0010 |
| Exact or approximate P value? | Approximate |
| P value summary | ** |
| Are means signif. different? (P < 0.05) | Yes |
| Number of groups | 4 |
| Friedman statistic | 16,20 |
|  |  |
| Data summary |  |
| Number of treatments (columns) | 4 |
| Number of subjects (rows) | 10 |

| Number of families | 1 |  |  |  |  |
| --- | --- | --- | --- | --- | --- |
| Number of comparisons per family | 3 |  |  |  |  |
| Alpha | 0,05 |  |  |  |  |
|  |  |  |  |  |  |
| Dunn's multiple comparisons test | Rank sum diff, | Significant? | Summary |  |  |
|  |  |  |  |  |  |
| Initial test vs. 2nd test | 9,000 | No | ns |  |  |
| Initial test vs. 3rd test | 21,00 | Yes | *** |  |  |
| Initial test vs. 4th test FLU | 2,000 | No | ns |  |  |
|  |  |  |  |  |  |
|  |  |  |  |  |  |
| Test details | Rank sum 1 | Rank sum 2 | Rank sum diff, | n1 | n2 |
|  |  |  |  |  |  |
| Initial test vs. 2nd test | 33,00 | 24,00 | 9,000 | 10 | 10 |
| Initial test vs. 3rd test | 33,00 | 12,00 | 21,00 | 10 | 10 |
| Initial test vs. 4th test FLU | 33,00 | 31,00 | 2,000 | 10 | 10 |

Two-way ANOVA of 4^th^ test

| Table Analyzed | rotations |  |  |  |  |
| --- | --- | --- | --- | --- | --- |
|  |  |  |  |  |  |
| Two-way ANOVA | Ordinary |  |  |  |  |
| Alpha | 0,05 |  |  |  |  |
|  |  |  |  |  |  |
| Source of Variation | % of total variation | P value | P value summary | Significant? |  |
| Interaction | 0,5924 | 0,5863 | ns | No |  |
| Row Factor | 3,716 | 0,1766 | ns | No |  |
| Column Factor | 7,282 | 0,0610 | ns | No |  |
|  |  |  |  |  |  |
| ANOVA table | SS | DF | MS | F (DFn, DFd) | P value |
| Interaction | 37,03 | 1 | 37,03 | F (1, 47) = 0,3005 | P = 0,5863 |
| Row Factor | 232,3 | 1 | 232,3 | F (1, 47) = 1,885 | P = 0,1766 |
| Column Factor | 455,1 | 1 | 455,1 | F (1, 47) = 3,694 | P = 0,0610 |
| Residual | 5545 | 47 | 123,2 |  |  |

Absolute turn angle (o)

LRN Control

| Initial test | 2nd test | 3rd test | 4th test control |
| --- | --- | --- | --- |
| 43380.0 | 18288.000000 | 32507.00 | 32725.000 |
| 65794.0 | 35801.000000 | 34040.00 | 34344.000 |
| 48576.0 | 41573.000000 | 41704.00 | 46454.000 |
| 49391.0 | 42783.000000 | 41608.00 | 45433.000 |
| 54925.0 | 32277.000000 | 42148.00 | 41387.000 |
| 43052.0 | 36433.000000 | 43842.00 | 49158.000 |
| 83840.0 | 50883.000000 | 28078.00 | 49553.000 |
| 46850.0 | 39031.000000 | 32300.00 | 30802.000 |
| 54720.0 | 26057.000000 | 31287.00 | 32781.000 |
| 85746.0 | 29451.000000 | 30974.00 | 30223.000 |
| 47632.0 | 27799.000000 | 22368.00 | 34953.000 |
| 47632.0 | 35066.000000 | 32625.00 | 33956.000 |

| Table Analyzed | LRN CTRL |
| --- | --- |
|  |  |
| Friedman test |  |
| P value | 0,0002 |
| Exact or approximate P value? | Approximate |
| P value summary | *** |
| Are means signif. different? (P < 0.05) | Yes |
| Number of groups | 4 |
| Friedman statistic | 19,20 |
|  |  |
| Data summary |  |
| Number of treatments (columns) | 4 |
| Number of subjects (rows) | 12 |

| Number of families | 1 |  |  |  |  |
| --- | --- | --- | --- | --- | --- |
| Number of comparisons per family | 3 |  |  |  |  |
| Alpha | 0,05 |  |  |  |  |
|  |  |  |  |  |  |
| Dunn's multiple comparisons test | Rank sum diff, | Significant? | Summary |  |  |
|  |  |  |  |  |  |
| Initial test vs. 2nd test | 24,00 | Yes | *** |  |  |
| Initial test vs. 3rd test | 24,00 | Yes | *** |  |  |
| Initial test vs. 4th test control | 16,00 | Yes | * |  |  |
|  |  |  |  |  |  |
|  |  |  |  |  |  |
| Test details | Rank sum 1 | Rank sum 2 | Rank sum diff, | n1 | n2 |
|  |  |  |  |  |  |
| Initial test vs. 2nd test | 46,00 | 22,00 | 24,00 | 12 | 12 |
| Initial test vs. 3rd test | 46,00 | 22,00 | 24,00 | 12 | 12 |
| Initial test vs. 4th test control | 46,00 | 30,00 | 16,00 | 12 | 12 |

LRN FLU

| Initial test | 2nd test | 3rd test | 4th test FLU |
| --- | --- | --- | --- |
| 45891.000 | 20527.000000 | 34844.000 | 28938.000 |
| 52134.000 | 36557.000000 | 44335.000 | 55442.000 |
| 59963.000 | 44042.000000 | 40470.000 | 40453.000 |
| 8488.000 | 37807.000000 | 37505.000 | 42784.000 |
| 81648.000 | 45118.000000 | 29304.000 | 46338.000 |
| 54703.000 | 41869.000000 | 13472.000 | 63176.000 |
| 42022.000 | 30847.000000 | 33497.000 | 38939.000 |
| 55288.000 | 32986.000000 | 26001.000 | 39598.000 |
| 84127.000 | 37089.000000 | 13364.000 | 68453.000 |
| 45787.000 | 27601.000000 | 31299.000 | 40534.000 |

| Table Analyzed | LRN FLU |
| --- | --- |
|  |  |
| Friedman test |  |
| P value | 0,0025 |
| Exact or approximate P value? | Approximate |
| P value summary | ** |
| Are means signif. different? (P < 0.05) | Yes |
| Number of groups | 4 |
| Friedman statistic | 14,28 |
|  |  |
| Data summary |  |
| Number of treatments (columns) | 4 |
| Number of subjects (rows) | 10 |

| Number of families | 1 |  |  |  |  |
| --- | --- | --- | --- | --- | --- |
| Number of comparisons per family | 3 |  |  |  |  |
| Alpha | 0,05 |  |  |  |  |
|  |  |  |  |  |  |
| Dunn's multiple comparisons test | Rank sum diff, | Significant? | Summary |  |  |
|  |  |  |  |  |  |
| Initial test vs. 2nd test | 17,00 | Yes | ** |  |  |
| Initial test vs. 3rd test | 18,00 | Yes | ** |  |  |
| Initial test vs. 4th test FLU | 5,000 | No | ns |  |  |
|  |  |  |  |  |  |
|  |  |  |  |  |  |
| Test details | Rank sum 1 | Rank sum 2 | Rank sum diff, | n1 | n2 |
|  |  |  |  |  |  |
| Initial test vs. 2nd test | 35,00 | 18,00 | 17,00 | 10 | 10 |
| Initial test vs. 3rd test | 35,00 | 17,00 | 18,00 | 10 | 10 |
| Initial test vs. 4th test FLU | 35,00 | 30,00 | 5,000 | 10 | 10 |

HRN Control

| Initial test | 2nd test | 3rd test | 4th test control |
| --- | --- | --- | --- |
| 23037.00000 | 272.00 | 39154.00 | 33707.000 |
| 50931.00000 | 38356.00 | 38522.00 | 39181.000 |
| 42573.00000 | 36983.00 | 35244.00 | 47685.000 |
| 5589.00000 | 34114.00 | 42176.00 | 42923.000 |
| 41715.00000 | 30809.00 | 11350.00 | 20122.000 |
| 69172.00000 | 36583.00 | 30955.00 | 33412.000 |
| 47348.00000 | 28591.00 | 28229.00 | 24447.000 |
| 64824.00000 | 26553.00 | 44385.00 | 40650.000 |
| 101828.00000 | 18079.00 | 32952.00 | 41106.000 |
| 48131.00000 | 36714.00 | 41205.00 | 42629.000 |
| 54983.00000 | 32686.00 | 28582.00 | 35363.000 |
| 27756.00000 | 44351.00 | 37171.00 | 33542.000 |
| 51861.00000 | 39113.00 | 37290.00 | 37540.000 |
| 31147.00000 | 34204.00 | 14254.00 | 30189.000 |

| Table Analyzed | HRN CTRL |
| --- | --- |
|  |  |
| Friedman test |  |
| P value | 0,0342 |
| Exact or approximate P value? | Approximate |
| P value summary | * |
| Are means signif. different? (P < 0.05) | Yes |
| Number of groups | 4 |
| Friedman statistic | 8,657 |
|  |  |
| Data summary |  |
| Number of treatments (columns) | 4 |
| Number of subjects (rows) | 14 |

| Number of families | 1 |  |  |  |  |
| --- | --- | --- | --- | --- | --- |
| Number of comparisons per family | 3 |  |  |  |  |
| Alpha | 0,05 |  |  |  |  |
|  |  |  |  |  |  |
| Dunn's multiple comparisons test | Rank sum diff, | Significant? | Summary |  |  |
|  |  |  |  |  |  |
| Initial test vs. 2nd test | 15,00 | No | ns |  |  |
| Initial test vs. 3rd test | 19,00 | Yes | * |  |  |
| Initial test vs. 4th test control | 10,00 | No | ns |  |  |
|  |  |  |  |  |  |
|  |  |  |  |  |  |
| Test details | Rank sum 1 | Rank sum 2 | Rank sum diff, | n1 | n2 |
|  |  |  |  |  |  |
| Initial test vs. 2nd test | 46,00 | 31,00 | 15,00 | 14 | 14 |
| Initial test vs. 3rd test | 46,00 | 27,00 | 19,00 | 14 | 14 |
| Initial test vs. 4th test control | 46,00 | 36,00 | 10,00 | 14 | 14 |

HRN FLU

| Initial test | 2nd test | 3rd test | 4th test FLU |
| --- | --- | --- | --- |
| 52277.00000 | 43531.000 | 13676.000 | 44184.000 |
| 66931.00000 | 47111.000 | 1232.000 | 49836.000 |
| 64835.00000 | 39220.000 | 1722.000 | 44907.000 |
| 43184.00000 | 44636.000 | 12661.000 | 38504.000 |
| 59121.00000 | 51562.000 | 25136.000 | 40753.000 |
| 67739.00000 | 48932.000 | 24493.000 | 55796.000 |
| 81108.00000 | 38947.000 | 26918.000 | 24227.000 |
| 67714.00000 | 49543.000 | 27826.000 | 26070.000 |
| 34061.00000 | 47178.000 | 22751.000 | 29874.000 |
| 43393.00000 | 49284.000 | 40611.000 | 50921.000 |

| Table Analyzed | HRN FLU |
| --- | --- |
|  |  |
| Friedman test |  |
| P value | 0,0004 |
| Exact or approximate P value? | Approximate |
| P value summary | *** |
| Are means signif. different? (P < 0.05) | Yes |
| Number of groups | 4 |
| Friedman statistic | 18,00 |
|  |  |
| Data summary |  |
| Number of treatments (columns) | 4 |
| Number of subjects (rows) | 10 |
|  |  |

| Number of families | 1 |  |  |  |  |
| --- | --- | --- | --- | --- | --- |
| Number of comparisons per family | 3 |  |  |  |  |
| Alpha | 0,05 |  |  |  |  |
|  |  |  |  |  |  |
| Dunn's multiple comparisons test | Rank sum diff, | Significant? | Summary |  |  |
|  |  |  |  |  |  |
| Initial test vs. 2nd test | 8,000 | No | ns |  |  |
| Initial test vs. 3rd test | 24,00 | Yes | **** |  |  |
| Initial test vs. 4th test FLU | 12,00 | No | ns |  |  |
|  |  |  |  |  |  |
|  |  |  |  |  |  |
| Test details | Rank sum 1 | Rank sum 2 | Rank sum diff, | n1 | n2 |
|  |  |  |  |  |  |
| Initial test vs. 2nd test | 36,00 | 28,00 | 8,000 | 10 | 10 |
| Initial test vs. 3rd test | 36,00 | 12,00 | 24,00 | 10 | 10 |
| Initial test vs. 4th test FLU | 36,00 | 24,00 | 12,00 | 10 | 10 |

Two-way ANOVA of 4^th^ test

| Table Analyzed | turn angle |  |  |  |  |
| --- | --- | --- | --- | --- | --- |
|  |  |  |  |  |  |
| Two-way ANOVA | Ordinary |  |  |  |  |
| Alpha | 0,05 |  |  |  |  |
|  |  |  |  |  |  |
| Source of Variation | % of total variation | P value | P value summary | Significant? |  |
| Interaction | 2,315 | 0,3098 | ns | No |  |
| Row Factor | 2,870 | 0,2588 | ns | No |  |
| Column Factor | 7,772 | 0,0667 | ns | No |  |
|  |  |  |  |  |  |
| ANOVA table | SS | DF | MS | F (DFn, DFd) | P value |
| Interaction | 8,670e+007 | 1 | 8,670e+007 | F (1, 47) = 1,058 | P = 0,3098 |
| Row Factor | 1,075e+008 | 1 | 1,075e+008 | F (1, 47) = 1,312 | P = 0,2588 |
| Column Factor | 2,911e+008 | 1 | 2,911e+008 | F (1, 47 = 3,554 | P = 0,0667 |
| Residual | 3,276e+009 | 47 | 8,191e+007 |  |  |
